# AMPK modulatory activity of olive–tree leaves phenolic compounds: Bioassay-guided isolation on adipocyte model and in silico approach

**DOI:** 10.1371/journal.pone.0173074

**Published:** 2017-03-09

**Authors:** Cecilia Jiménez-Sánchez, Mariló Olivares-Vicente, Celia Rodríguez-Pérez, María Herranz-López, Jesús Lozano-Sánchez, Antonio Segura-Carretero, Alberto Fernández-Gutiérrez, José Antonio Encinar, Vicente Micol

**Affiliations:** 1 Department of Analytical Chemistry, University of Granada. Granada, Spain; 2 Research and Development of Functional Food Centre (CIDAF), PTS, Granada, Spain; 3 Instituto de Biología Molecular y Celular (IBMC), Miguel Hernández University (UMH), Elche, Alicante, Spain; 4 CIBER: CB12/03/30038, Fisiopatología de la Obesidad y la Nutrición, CIBERobn, Instituto de Salud Carlos III (ISCIII), Palma de Mallorca, Spain; Dokuz Eylul Universitesi, TURKEY

## Abstract

**Scope:**

Olive-tree polyphenols have demonstrated potential for the management of obesity-related pathologies. We aimed to explore the capacity of Olive-tree leaves extract to modulate triglyceride accumulation and AMP-activated protein kinase activity (AMPK) on a hypertrophic adipocyte model.

**Methods:**

Intracellular triglycerides and AMPK activity were measured on the hypertrophic 3T3-L1 adipocyte model by AdipoRed and immunofluorescence microscopy, respectively. Reverse phase high performance liquid chromatography coupled to time-of-flight mass detection with electrospray ionization (RP-HPLC-ESI-TOF/MS) was used for the fractionation of the extract and the identification of the compounds. In-silico molecular docking of the AMPK alpha-2, beta and gamma subunits with the identified compounds was performed.

**Results:**

Olive-tree leaves extract decreased the intracellular lipid accumulation through AMPK-dependent mechanisms in hypertrophic adipocytes. Secoiridoids, cinnamic acids, phenylethanoids and phenylpropanoids, flavonoids and lignans were the candidates predicted to account for this effect. Molecular docking revealed that some compounds may be AMPK-gamma modulators. The modulatory effects of compounds over the alpha and beta AMPK subunits appear to be less probable.

**Conclusions:**

Olive-tree leaves polyphenols modulate AMPK activity, which may become a therapeutic aid in the management of obesity-associated disturbances. The natural occurrence of these compounds may have important nutritional implications for the design of functional ingredients.

## Introduction

Obesity is a multifactorial complex disease of global significance. This disease is defined by excess adipose mass and adipose tissue expansion, which occurs through adipocyte hypertrophy and hyperplasia [1]. Importantly, adipocyte size is a major determinant of obesity in adults [2]. Adipose tissue is a key energy storage organ, and adipose endocrine function is critical to the overall energy balance and homeostasis with adipocyte-derived pro- and anti-inflammatory adipokines playing key roles [3]. When the production and secretion of proinflammatory adipokines prevails, systemic inflammation, insulin resistance and obesity-related metabolic disorders arise [4].

Recently, AMP-activated protein kinase (AMPK) has been revealed to be an important regulator of cellular energy homeostasis. AMPK is a sensor of the cellular energy status that directs metabolic adaptation to support cellular growth and survival, restoring energy homeostasis. AMPK is involved in the regulation of carbohydrate and lipid metabolism, resulting in the inhibition of ATP-consuming anabolic pathways, including FA (fatty acid) synthesis, cholesterol and isoprenoid synthesis, hepatic gluconeogenesis and mTOR (mammalian target of rapamycin)-mediated protein translation. In parallel, AMPK activation stimulates ATP production by increasing FA oxidation, muscle glucose transport, mitochondrial biogenesis and caloric intake [5,6]. It also plays a major role in hormonal signaling and is a central node of such signaling pathways. It can regulate the endocrine system, and at the same time, its activity is regulated by a number of hormones and cytokines (adipokines) such as leptin, interleukin-6, resistin, ghrelin, and adiponectin. In addition, it controls the appetite through a neuroendocrine system that makes it a key regulator of energy metabolism at the whole body level [7].

AMPK is activated by phosphorylation at Thr172, which is modulated by the binding of AMP. Although allosteric activation is only caused by AMP, it has recently been found that similar effects on phosphorylation and dephosphorylation can also be produced by ADP [8]. Specifically, AMPK is phosphorylated by upstream kinases. The primary upstream AMPK kinase is the liver kinase B1 (LKB1), a product of a tumor suppressor gene, which provides anti-tumor functions through the direct phosphorylation of AMPK Thr172 *in vitro* and *in vivo* [9,10]. Secondly, Calcium/calmodulin-dependent protein kinase 2 (CaMKKβ), triggers the activation of AMPK in response to increases in cell Ca^2+^ [11]. Increases in the Ca^2+^ influx usually accompany such processes as the activation of motor proteins and messages of increased energy consumption. Thus, this activation mechanism anticipates ATP deficiency before it has occurred [12]. The classical pathways through which AMPK is activated by upstream kinases or Ca^2+^ are now becoming well understood, although the understanding of how phosphatases dephosphorylate the protein in Thr172 remains incomplete [13]. Although changes in the AMP, ATP, or Ca^2+^ levels are triggered by metabolic stresses, recent work suggests that AMPK can also be switched on by numerous plant-derived phenolic compounds [14–17]. To date, sufficient evidence has been accumulated to support that phenolic compounds of *Olea europaea* might be able to activate AMPK pathways in cancer cell lines through the AMPK/mTOR axis [18,19]. These findings led us to postulate that these compounds could also have important implications in metabolic stress-related disorders such as obesity through AMPK-dependent mechanisms.

In this context, we have studied the capacity of an Olive-tree leaves extract to modulate triglyceride accumulation and AMPK activation using the well-established 3T3-L1 adipocyte model. The characterization of the phenolic extract was achieved using RP-HPLC-ESI-TOF/MS, and semi-preparative fractionation was performed to identify putative candidates for the attributed biological activity. A molecular docking approach was utilized to obtain the free energy variation of the molecular structures of the identified compounds on the AMPK alpha-2, beta and gamma subunits to search for direct AMPK interaction. Finally, a correlation between the most active Olive-tree leaves compounds in the cellular model and those bearing lower free energy values is proposed for the rational understanding of AMPK modulation by the extract.

## Materials and methods

### Materials

For the semi-preparative fractionation of the Olive-tree leaves extract, all chemicals were of analytical reagent grade and were used as received. Methanol used for the extraction was purchased from Panreac (Barcelona, Spain). Acetic acid and acetonitrile for semi-preparative HPLC were purchased from Fluka and Sigma-Aldrich (Steinheim, Germany), respectively. Water was purified by a Milli-Q system from Millipore (Bedford, MA, USA). 3T3-L1 mouse embryo fibroblasts were purchased from the American Type Culture Collection (Manassas, VA, USA). Dexamethasone (DEX), 3-isobutyl-1-methylxanthine (IBMX), insulin, penicillin—streptomycin, calf serum, fetal bovine serum (FBS) (both being HyClone), paraformaldehyde solution, and Triton X-100 were obtained from Sigma-Aldrich (Madrid, Spain). Dulbecco’s modified Eagle’s medium (DMEM) was purchased from Gibco (ThermoFisher Scientific, Waltham, MA, USA). Polyvinyldifluoride (PVD) filters, 0.22 μm, were obtained from Millipore (Bedford, MA, USA), and Dulbecco’s phosphate buffered saline (PBS) was obtained from Sigma-Aldrich (St. Louis, MO, USA). The staining AdipoRed Assay Reagent was obtained from Lonza (Walkersville, MD USA).

### Preparation of the Olive-tree leaves extract

Olive-tree leaves (*Olea europaea*) from the ‘Arbequina’ cultivar were used in this study. They were air-dried in the laboratory, and sample extraction was performed as described elsewhere [20]. Briefly, dry leaves (5 g) were crushed and extracted via Ultra—Turrax T18 basic reagent (IKA, Staufen, Germany) using 300 mL of MeOH/H_2_O (80/20). After solvent evaporation, the extracts were reconstituted with MeOH/H_2_O (50/50) to achieve a concentration of 50 mg/mL. Three extraction replicates were processed.

### Analytical characterization of the Olive-tree leaves extract and isolated fractions

The Olive-tree leaves extract was analytically characterized by RP-HPLC-ESI-TOF/MS, performed in an Agilent 1200-HPLC system (Agilent Technologies, Waldbronn, Germany) of the Series Rapid Resolution equipped with a vacuum degasser, autosampler, a binary pump, and a UV-vis detector. The chromatographic separation was carried out as reported elsewhere [21]. The extract and the fractions were injected at a concentration of 1 mg/mL.

The compounds separated were monitored with a mass-spectrometry detector. MS was performed using a microTOF (Bruker Daltonik, Bremen, Germany), which was equipped with an ESI interface (model G1607A from Agilent Technologies, Palo Alto, CA, USA) operating in the negative-ion mode. The optimum values of the source and transfer parameters were described elsewhere [22]. The accurate mass data for the molecular ions were processed using the software Data Analysis 3.4 (Bruker Daltonik).

### Semi-preparative fractionation of the Olive-tree leaves extract

Semi-preparative fractionation of the Olive-tree leaves extract was achieved using a Gilson preparative HPLC system (Gilson, Middleton, WI, USA) equipped with a binary pump (model 331/332), an automated liquid-handling solution (model GX-271), and a UV-Vis detector (model UV-Vis 156). The extract was fractionated at room temperature. A 250 mm x 10 mm i.d., 5 μm Phenomenex RP-C18 column was used to separate the compounds. The mobile phases consisted of acetic acid 0.5% (A) and acetonitrile (B). The following multi-step linear gradient was applied: 0 min, 5% B; 5 min, 15% B; 53 min, 27% B; 54 min, 28% B; 60 min, 100% B; 65 min, 100% B; 70 min, 5% B; 75 min, 5% B. The injection volume was 500 μL and the flow rate used was set at 10 mL/min. The compounds separated were monitored with UV-Vis (240 and 280 nm) and MS, using the time-of-flight mass spectrometer detector microTOF (Bruker Daltonik, Bremen, Germany), as reported in the previous section.

### Cell culture and treatment and intracellular lipid quantitation

The 3T3-L1 preadipocyte cell line was cultured and differentiated as described elsewhere [23]. Hypertrophied adipocytes were obtained using high glucose medium containing insulin (4.5 mg/mL) after differentiation [24]. Under these conditions, cells become hypertrophic adipocytes, a cell model characterized by a high level of cytoplasmic lipid accumulation, insulin-resistance, and exacerbated oxidative stress, a situation reasonably similar to that of obese adipose tissue [16,24]. On day 18, cells were treated with the respective extract or fractions, which were dissolved in 10% FBS/high glucose (4.5 mg/mL) DMEM medium with 1 μM insulin and maintained for 48 h. For cell treatments, extract or fractions were reconstituted in cell culture medium plus DMSO at a maximum final concentration of 0.3% v/v after solvent evaporation. The crystal violet assay was performed as reported to dismiss the possible cytotoxic effects of the extract/fractions at the working concentrations [24,25]. Quantification of intracellular lipid droplets in hypertrophic adipocytes was performed as reported [24] using the staining AdipoRed Assay Reagent and the Cytation 3 cell imaging multi-mode microplate reader (Biotek Instruments, Winooski, VT, USA).

### Quantification of the AMPK and pAMPK levels

For AMPK and phospho-AMPK (pAMPK) detection, an immunofluorescence assay was carried out. Cells were washed with PBS, fixed for 15 min in 4% paraformaldehyde, permeabilized in 0.25% Triton X-100 for 5 min, and washed with PBS. After blocking in 4% goat serum at room temperature for 1 h, the cells were washed and incubated overnight at 4°C with mouse monoclonal to AMPK alpha 1 + AMPK alpha 2 antibodies (Abcam, Cambridge, UK) or rabbit monoclonal phospho-AMPKα (Thr172) antibodies (Cell Signaling Technology, Danvers, MA, USA). Cells were washed 3 times with PBS and incubated at ambient temperatures for 6 hours with Hoechst staining (2.5 μg/mL) together with each corresponding polyclonal secondary antibody, goat anti-rabbit IgG CF 594, or goat anti-mouse polyvalent immunoglobulins (G,A,M)-FITC, all from Sigma-Aldrich (St. Louis, MO, USA). Cells were washed 3 times with PBS and read with a Cytation 3 cell imaging multi-mode microplate reader (Biotek Instruments, Winooski, VT, USA). AMPK was detected by measuring the fluorescence with excitation at 490 nm and emission at 520 nm for AMPK and with excitation at 590 nm and emission at 620 nm for pAMPK. To ensure that the extracts/fractions were not cytotoxic, the cell count was performed by taking microphotographs of the Hoechst-stained cells at 4x, using the DAPI imaging filter cube.

### Molecular docking procedures

To date, several crystal structures of the three AMPKs have been solved and deposited into the Protein Data Bank, many of them with their inhibitors that delimitate their binding site. For AMPK catalytic subunit alpha-2 (UniProt P54646), the following structures have been used as a receptor in the molecular docking experiments for AMPK beta subunit (UniProt Q9Y478): 4CFE, 4CFF, 4ZHX, 5EZV. Finally, for the AMPK gamma subunit (UniProt P54619), several crystal structures were chosen: 2UV4, 2UV5, 2UV6, 2UV7, 4CFE, 4CFF, 4RER, 4REW, and 4ZHX. Prior to initiating the docking procedure, the protein (receptor) and ligand structures should be prepared [26]. A grid with the dimensions of 22 x 22 x 22 points was centered to ensure coverage of the binding site of the structure. AutoDock/Vina was set up on a Microsoft Windows 10 system using configuration files, including the usage of two CPUs and an effectiveness of 20.

### Statistical analysis

Values are represented as the mean ± standard deviation (S.D.) of the mean. The values were subjected to statistical analysis (one-way ANOVA, and Dunett’s test for multiple comparisons/non-parametric approaches). The differences were considered to be statistically significant at *p* < 0.05. All analyses were performed using Graph Pad Prism 6 (GraphPad Software, Inc. La Jolla, CA, USA). *p < 0.05, **p < 0.01 and ***p < 0.001 on the bars indicate statistically significant differences compared to the control, unless otherwise stated. All cellular measurements were derived from three independent experiments. Each experiment was performed in sextuplicate, unless otherwise specified.

## Results

### Characterization of the Olive-tree leaves extract by RP-HPLC-ESI-TOF

**Fig 1:**
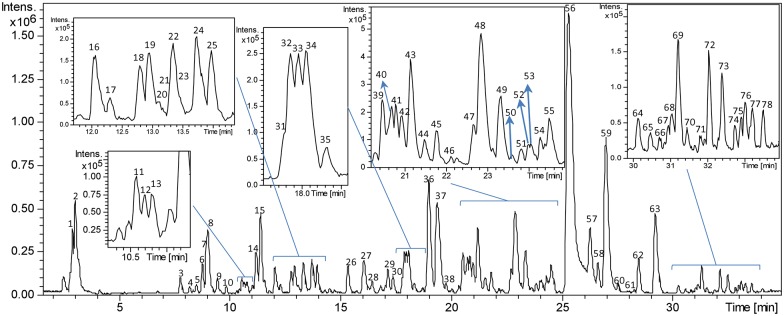
HPLC-MS characterization of Olive-tree leaves extract. Base peak chromatogram (BPC) of the Olive-tree leaves extract in the negative ion mode obtained by RP-HPLC-ESI-TOF/MS, in which the peaks are identified by numbers 1–78 according to the elution order.

The Olive-tree leaves extract obtained as described in the materials and methods section was characterized using the RP-HPLC-ESI-TOF/MS methodology. The identification was performed by comparing the retention times from the MS data provided by the TOF analyzer and those from the literature (Fig 1).

The chromatographic profile obtained by RP-HPLC-ESI-TOF/MS showed several peaks, of which a total of 78 were tentatively identified. Compound numbers were assigned according to their elution order.

As shown in Table 1, most of the compounds belonged to the phenolic compound classes, specifically to the iridoid (most of them being secoiridoids), phenylethanol derivative, cinnamic acid, phenylpropanoid, coumarin, flavonoid, and lignan subclasses. The chemical structures of the identified compounds are included in S1 Fig.

**Table 1:** Relevant mass data of the proposed compounds detected in the Olive-tree leaves extract analyzed by RP-HPLC-ESI-TOF/MS. From left to right: peak number, retention time, calculated *m/z*, calculated *m/z*, molecular formula, error (ppm), millisigma value, proposed compound; and reference and matrix in which the proposed compound has been previously described.

Pk	RT (min)	*m/z* exp.	*m/z* calc.	Mol. formula	Error (ppm)	mSigma	Proposed compound	Reference	Matrix
1	2.86	341.1086	341.1089	C_12_H_22_O_11_	0.9	89.4	Sucrose	[49]	Olive-tree leaves
2	3.096	191.057	191.0561	C_7_H_12_O_6_	4.8	14.8	Quinic acid	[21]	Olive-tree leaves
3	7.811	375.1296	375.1297	C_16_H_24_O_10_	0.3	9.2	(Epi)loganic acid isomer1	[50]	Olive pomace
4	8.229	389.1073	389.1089	C_16_H_22_O_11_	4.1	9.9	Oleoside/ Secologanoside isomer 1	[21,51]	Olive-tree leaves
5	8.631	389.1074	389.1089	C_16_H_22_O_11_	4	12.3	Oleoside/ Secologanoside isomer 2	[21,51]	Olive-tree leaves
6	8.797	315.1086	315.1085	C_14_H_20_O_8_	0.1	16.5	Hydroxytyrosol-glucoside isomer 1	[51–53]	Olive-tree leaves
7	8.849	315.1101	315.1085	C_14_H_19_O_8_	5	33.3	Hydroxytyrosol-glucoside isomer 2	[51–53]	Olive-tree leaves
8	9.048	389.1112	389.1089	C_16_H_22_O_11_	5.7	13.7	Oleoside/ Secologanoside isomer 3	[21,51]	Olive-tree leaves
9	9.466	375.1299	375.1297	C_16_H_24_O_10_	0.5	20.3	(Epi)loganic acid isomer 2	[50,53]	Olive pomace
10	9.868	153.0543	153.0557	C_8_H_10_O_3_	9.3	16	Hydroxytyrosol	[21,52,53]	Olive-tree leaves
11	10.353	299.1136	299.1121	C_14_H_20_O_7_	5	23.8	Tyrosol glucoside	[54]	Olive-tree leaves
12	10.486	341.0875	341.0878	C_15_H_18_O_9_	1	5	Caffeoylglucoside	[55]	Olive mill wastewater
13	10.687	339.0702	339.0722	C_15_H_16_O_9_	5.8	12.2	Esculin	[53]	Olive-tree leaves
14	11.189	403.1246	403.1246	C_17_H_24_O_11_	0.2	19.8	Elenolic acid glucoside/methyloleoside isomer 1	[21,51]	Olive-tree leaves
15	11.423	389.1106	389.1089	C_16_H_22_O_11_	4.2	14.6	Oleoside/ Secologanoside isomer 4	[21,51]	Olive-tree leaves
16	12.125	403.1241	403.1246	C_17_H_24_O_11_	1.3	11.8	Elenolic acid glucoside/methyloleoside isomer 2	[21,51]	Olive-tree leaves
17	12.309	325.0918	325.0918	C_15_H_18_O_8_	3.5	5.7	*p*-oumaric acid glucoside	[56]	*Ligustrum purpurascens* leaves (Oleaceae)
18	12.794	519.1727	519.1872	C_22_H_32_O_14_	2.7	50.6	Unknown	–	–
19	12.878	401.1476	401.1453	C_18_H_26_O_10_	3.7	21.6	Unknown	[51]	Olive-tree leaves
20	12.944	593.1524	593.1512	C_27_H_30_O_15_	2	9.2	Unknown	–	–
21	13.246	387.2001	387.2024	C_19_H_32_O_8_	6	15.2	Unknown	–	–
22	13.329	403.1258	403.1246	C_17_H_24_O_11_	2.9	20.5	Elenolic acid glucoside/methyloleoside isomer 3	[21,51]	Olive-tree leaves
23	13.43	537.2002	537.1978	C_26_H_34_O_12_	-4.5	3.3	Olivil glucoside	[53]	Olive stem
24	13.684	377.1461	377.1453	C_19_H_22_O_8_	2.2	10.9	Oleuropein aglycone	[49,52]	Olive-tree leaves
25	14.099	609.1486	609.1461	C_27_H_30_O_16_	4.1	10.4	Glucosyl rhamnosylquercetin (rutin) isomer 1	[54]	Olive-tree leaves
26	15.352	403.1222	403.1246	C_17_H_24_O_11_	5.8	13.9	Elenolic acid glucoside methyloleoside isomer 4	[21,51]	Olive-tree leaves
27	16.055	415.1628	415.161	C_19_H_28_O_10_	4.4	21	Phenethyl primeveroside	[57]	Isolated from olive cells
28	16.391	403.1958	403.1974	C_19_H_32_O_9_	3.8	24.9	Ethyl-glucopyranosyloxy-oxopropyl-cyclohexaneacetic acid	[58]	Olive-tree leaves
29	17.445	511.2374	511.2396	C_22_H_40_O_13_	4.3	6.9	Unknown	–	–
30	17.476	525.1611	525.1614	C_24_H_30_O_13_	0.4	7.6	Demethyloleuropein	[53]	Olive-tree leaves
31	17.794	701.2279	701.2298	C_31_H_42_O_18_	2.7	8.1	Oleuropein glucoside/neonuezhenide isomer 1	[52]	Olive-tree leaves
32	17.843	555.1744	555.1719	C_25_H_32_O_14_	4.4	19.9	Hydroxyoleuropein isomer 1	[21]	Olive-tree leaves
33	17.877	609.1476	609.1461	C_27_H_30_O_16_	2.5	20.9	Glucosyl rhamnosylquercetin (rutin) isomer 2	[54]	Olive-tree leaves
34	17.795	593.154	593.1512	C_27_H_30_O_15_	4.7	29.2	Luteolin rutinoside/luteolin neohesperidoside/apigenin diglucoside	[21,53]	Olive-tree leaves
35	18.799	375.1426	375.1449	C_20_H_24_O_7_	6.2	14.8	Olivil	[53]	Olive-tree leaves
36	18.982	623.2004	623.1981	C_29_H_36_O_15_	6.2	8.4	Verbascoside	[21,49]	Olive-tree leaves
37	19.601	447.0964	447.0933	C_21_H_20_O_11_	6.9	61.7	Luteolin glucoside isomer 1	[21,51,52]	Olive-tree leaves
38	19.735	477.1393	477.1402	C_23_H_26_O_11_	2	26.7	Unknown	–	–
39	20.486	555.1722	555.1719	C_25_H_32_O_14_	0.5	23.9	Hydroxyoleuropein isomer 2	[21]	Olive-tree leaves
40	20.703	701.2329	701.2298	C_31_H_42_O_18_	-4.4	16.5	Oleuropein glucoside/neonuezhenide isomer 2	[52]	Olive-tree leaves
41	20.806	577.1582	577.1563	C_27_H_30_O_14_	-3.3	33.7	Apigenin rutinoside/apigenin neohesperidoside	[21,53]	Olive-tree leaves
42	20.937	701.2318	701.2298	C_31_H_42_O_18_	-2.8	15.9	Oleuropein glucoside/neonuezhenide isomer 3	[52]	Olive-tree leaves
43	20.772	701.2329	701.2298	C_31_H_42_O_18_	-4.4	16.5	Oleuropein glucoside/neonuezhenide isomer 4	[52]	Olive-tree leaves
44	21.489	701.2374	701.2298	C_31_H_42_O_18_	-10.7	28.2	Oleuropein glucoside/neonuezhenide isomer 5	[52]	Olive-tree leaves
45	21.756	607.166	607.1668	C_28_H_32_O_15_	1.4	17.3	Diosmetin rhamnoside glucoside (diosmin) isomer 1	[53]	Olive-tree leaves
46	22.275	607.1668	607.1668	C_28_H_32_O_15_	0	26.8	Diosmetin rhamnoside glucoside (diosmin) isomer 2	[53]	Olive-tree leaves
47	22.642	431.0977	431.0984	C_21_H_20_O_10_	1.5	22.8	Apigenin glucoside	[21,49]	Olive-tree leaves
48	22.826	447.0982	447.0933	C_21_H_20_O_11_	-11.1	38.6	Luteolin glucoside isomer 2	[21,51,52]	Olive-tree leaves
49	23.311	461.1112	461.1089	C_22_H_22_O_11_	-4.9	18.6	Diosmetin glucoside	[54]	Olive-tree leaves
50	23.463	491.155	491.1559	C_24_H_28_O_11_	1.8	5.6	Calceolarioside isomer 1	[53]	Olive-tree leaves
51	23.779	541.1925	541.1927	C_25_H_34_O_13_	0.2	16.5	Hydro-oleuropein	[59]	Olives and olive oil-derived matrices
52	24.049	491.1535	491.1559	C_24_H_28_O_11_	4.9	12.1	Calceolarioside isomer 2	[53]	Olive-tree leaves
53	23.948	539.1778	539.177	C_25_H_32_O_13_	-1.4	492.2	Oleuropein/oleuroside isomer 1	[21,49,52]	Olive-tree leaves
54	24.247	569.1906	569.1876	C_26_H_34_O_14_	-5.4	7.6	Methoxyoleuropein	[51]	Olive-tree leaves
55	24.532	447.0951	447.0933	C_21_H_20_O_11_	-4	4.4	Luteolin glucoside isomer 3	[21,51,53]	Olive-tree leaves
56	25.152	539.1808	539.177	C_25_H_32_O_13_	-7	25.3	Oleuropein/oleuroside isomer 2	[21,49,52]	Olive-tree leaves
57	26.221	539.1804	539.177	C_25_H_32_O_13_	-6.3	35	Oleuropein/oleuroside isomer 3	[21,49,52]	Olive-tree leaves
58	26.756	537.1571	537.1614	C_25_H_30_O_13_	8	223	Unknown	–	–
59	26.923	539.1793	539.177	C_25_H_32_O_13_	-4.3	7.1	Oleuropein/oleuroside isomer 4	[21,49,52]	Olive-tree leaves
60	27.441	557.2363	557.224	C_26_H_38_O_13_	-4.3	8.9	[Dimetyl hydroxy octenoyloxi] secologanoside isomer 1	[21]	Olive-tree leaves
61	28.16	793.2821	793.2866	C_45_H_46_O_13_	-1.9	59.1	Unknown	–	-
62	28.378	601.2156	601.2138	C_27_H_38_O_15_	-3.1	6	Piperchabaoside/(epi)frameroside)/ligustalisode dimethylacetal	[60]	*Ligustrum ovalifolium*
63	29.13	523.1802	523.1821	C_25_H_32_O_12_	3.7	6.1	Ligstroside isomer 1	[21,49,51,52]	Olive-tree leaves
64	30.15	593.1285	593.1301	C_30_H_26_O_13_	2.7	12.9	Unknown	-	-
65	30.485	557.2276	557.224	C_26_H_38_O_13_	6.6	18.7	Ddimethyl hydroxy octenoyloxi secologanoside isomer 2	[21]	Olive-tree leaves
66	30.719	523.18	523.1821	C_25_H_32_O_12_	3.9	31.6	Ligstroside isomer 2	[21,49,51,52]	Olive-tree leaves
67	30.936	553.1948	553.1927	C_26_H_34_O_13_	-3.9	19	Oleuropein methyl ether	[61]	Olive wood
68	31.02	539.178	539.177	C_25_H_32_O_13_	1.7	4.4	Oleuropein/oleuroside isomer 5	[21,49]	Olive-tree leaves
69	31.204	285.0412	285.0405	C_15_H_10_O_6_	-2.7	19.2	Luteolin	[21,51,53]	Olive-tree leaves
70	31.454	301.036	301.0354	C_15_H_10_O_7_	-2.1	5.9	Quercetin	[51,53]	Olive-tree leaves
71	31.789	613.195	613.1927	C_31_H_34_O_13_	-3.9	13.3	Resinoside	[62]	Eucalyptus leaves
72	32.259	615.2125	615.2083	C_31_H_36_O_13_	-6.8	6.5	Unknown	–	–
73	32.726	327.2178	327.2177	C_18_H_32_O_5_	-0.3	14	Unknown	–	–
74	32.876	331.2502	331.249	C_18_H_36_O_5_	3.6	16.5	Trihydroxystearic acid	[63]	Olive-tree leaves
75	32.96	269.0479	269.0455	C_15_H_10_O_5_	-8.7	9.5	Apigenin	[52]	Olive-tree leaves
76	33.196	329.2339	329.2333	C_18_H_34_O_5_	-1.8	28.9	Trihydroxy-octadecenoic acid	[64]	*Artemisia vulgaris* leaf
77	33.38	285.0417	285.0405	C_15_H_10_O_6_	-4.5	8	Unknown	–	–
78	33.715	287.2206	287.2228	C_16_H_32_O_4_	7.6	4.6	Dihydroxyhexadecanoic acid	[63]	Olive-tree leaves

### The effects of *Olea euroapea* leaf extract on triglyceride accumulation and AMPK activity modulation in 3T3-L1 hypertrophic adipocytes

**Fig 2:**
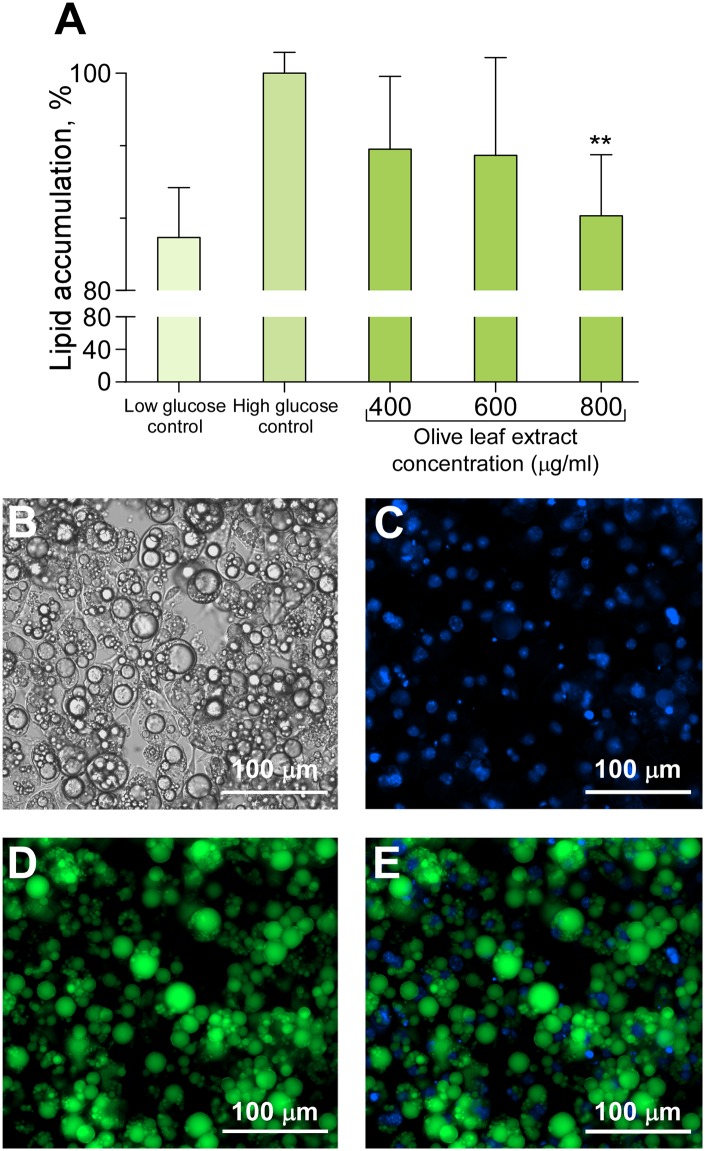
Intracellular triglyceride accumulation inhibitory effect of the complete Olive-tree leaves extract in 3T3-L1 hypertrophic adipocytes. (A) Quantitative assessment of lipid vesicles in hypertrophic adipocytes incubated with 400, 600, or 800 μg/mL of Olive-tree leaves extract and compared to the control in a high glucose medium. Values have been normalized with respect to the control incubated in a high glucose medium. ** p<0.01 indicates significant differences compared to the control. Representative microphotographs for the qualitative assessment of 3T3-L1 lipid droplets: hypertrophic adipocytes differentiated for 22 days, phase contrast (panel B), same cells stained for triglycerides in lipid droplets (green, panel D) and nuclei stained with DAPI to show the localization of nuclear DNA (blue, panel C). Superimposed lipid droplets and cellular nucleus (panel E).

Hypertrophic and insulin resistant adipocytes were treated for 48 hours with different concentrations of the crude extract. After 48 h, the quantification of intracellular lipids was measured with the probe AdipoRed. Cells treated with 400, 600, and 800 μg/mL of the extract decreased triglyceride accumulation by 93, 92, and 86%, respectively (Fig 2), but statistically significant differences compared with the non-treated cells maintained in high glucose medium were found only at 800 μg/mL of the extract (*p*<0.01). Microscopic observation of cells treated with 800 μg/mL of the extract revealed a significant reduction in the intracellular triglyceride accumulation.

**Fig 3:**
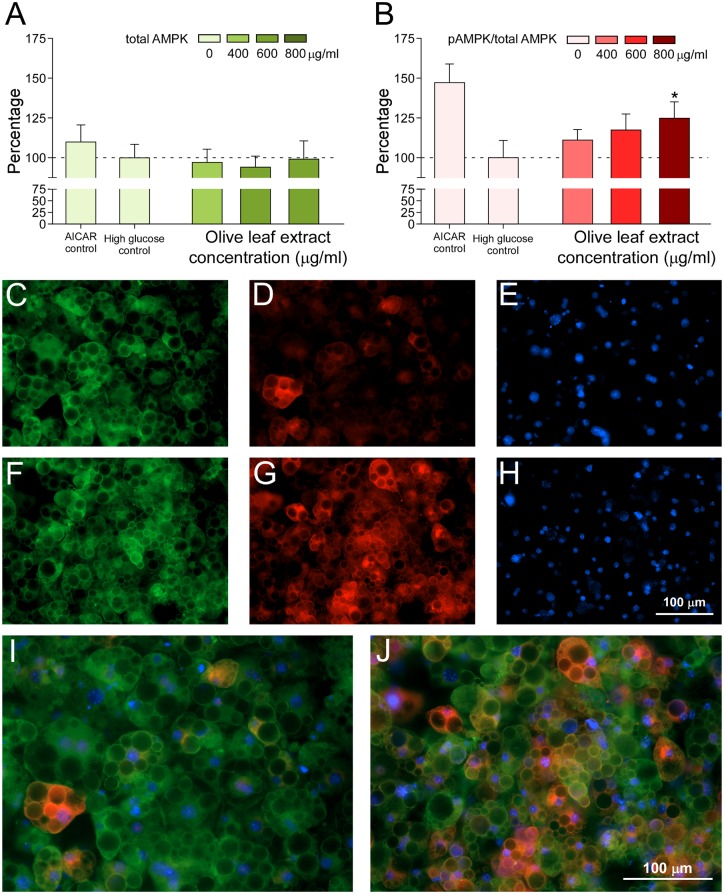
Olive-tree leaves extract activates AMPK in hypertrophic adipocytes. Measurement of the total AMPK expression levels (A) and the activation/inhibition rate (%), measured as the ratio pAMPK/AMPK, (B) Immunofluorescence microscopy of hypertrophic adipocytes treated with 400, 600, and 800 μg/mL of the olive-tree leaves extract and incubated in high glucose medium. Values were normalized with respect to the high glucose control. With comparative aims, the positive control 5-Aminoimidazole-4-carboxamide ribonucleotide (AICAR) has also been included. *p<0.05 indicates significant differences compared to the control incubated in high glucose medium. Representative microphotographs taken with a fluorescence microscope at 20x: control cells incubated in high glucose medium (total AMPK, green fluorescence, C; pAMPK, red fluorescence, D; nuclei, blue fluorescence, E; superimposed image, I) *vs*. cells incubated with 800 μg/mL of olive-tree leaves extract (total AMPK, F; pAMPK, G; nuclei, H; superimposed image, J).

With the aim of unveiling the possible molecular mechanisms of the reduction of intracellular lipid accumulation, we studied the effects of the Olive-tree leaves crude extract on the activation of AMPK through the phosphorylation of Thr172. After 48 hours of treatment with increasing concentrations of the extract, the levels of AMPK and pAMPK at Thr 172 (pAMPK) were quantified by immunofluorescence (Fig 3). No significant changes in the total AMPK protein levels were detected (Fig 3A), while the phosphorylation of the protein showed a dose-response behavior upon the increase of the Olive-tree leaves extract concentration, exhibiting significant differences at 800 μg/mL compared to the high glucose-treated control cells (*p*<0.05). At this concentration, the Olive-tree leaves extract promoted an important AMPK activation, considering that one observed with the AMPK activator AICAR, an AMP synthetic analog (Fig 3B). Microscopic observation of the treated cells also revealed a significant increase in the fluorescence intensity corresponding to pAMPK levels in cells treated with the complete extract at 800 μg/mL (Fig 3G) compared to the control (Fig 3D).

### Bio-assay guided isolation of AMPK modulators derived from the Olive-tree leaves extract by semipreparative liquid chromatography

To delimit the bioactive compounds responsible for the observed AMPK activation of the Olive-tree leaves extract, we developed a semi-preparative HPLC-ESI-TOF/MS methodology to fractionate the extract according to the UV and MS information. Twenty-eight fractions were obtained to be analyzed for AMPK activation (S2 Fig).

**Fig 4:**
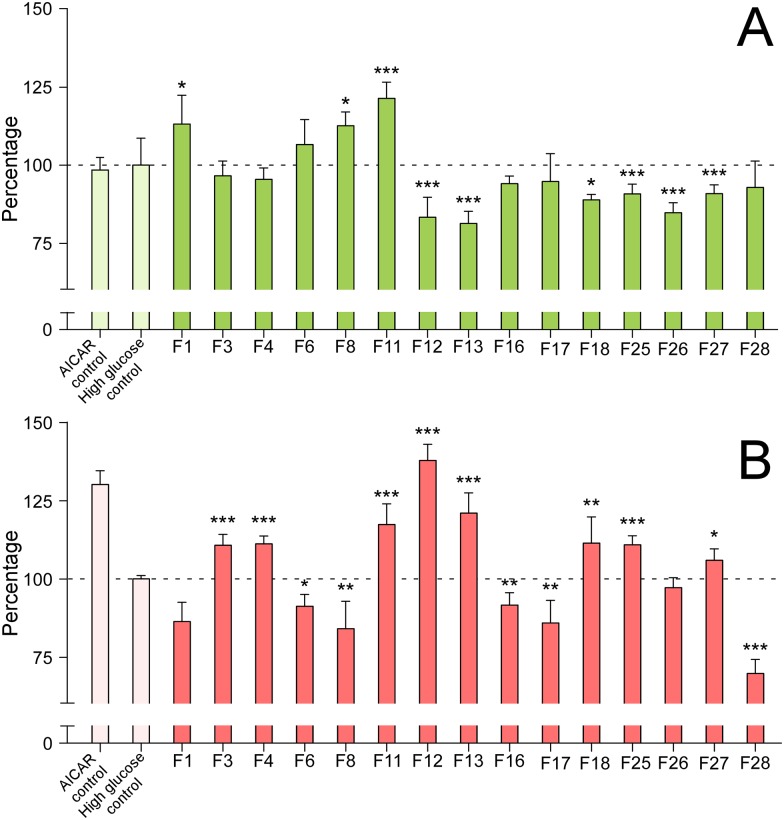
Modulation of AMPK expression and/or activity by Olive-tree leaves fractions. Measurement of the total AMPK expression levels (A) and the activation/inhibition rate (%), measured as the ratio pAMPK/AMPK (B) of the selected olive-tree leaves extract fractions in 3T3-L1 hypertrophic adipocytes. The modulatory effects on the AMPK expression levels and the effects on the pAMPK/AMPK ratio were quantified by immunofluorescence microscopy at a concentration of 400 μg/mL in cells incubated in high glucose medium. Values are normalized with respect to the high glucose control. With comparative aims, the positive control 5-Aminoimidazole-4-carboxamide ribonucleotide (AICAR) was also included. *, **, and *** indicate significant differences with respect to the control incubated in high glucose medium (*p*<0.05, *p*<0.01, and *p*<0.001, respectively).

To study which individual compounds were responsible for the mentioned AMPK activation/inhibition, fractions were incubated for 48 hours at a concentration of 400 μg/mL. The levels of AMPK and pAMPK were quantified by immunofluorescence as described in the Methods (Fig 4). Some fractions, such as F1, F8 and F11, exhibited increased levels of total AMPK expression, while some others showed a weak but significant decrease in the AMPK expression levels compared to the controls, i.e., F12, F13, F18, F25, F26 and F27, (Fig 4A). In addition, some fractions showed a significant AMPK activation (F3, F4, F11, F12, F13, F18, F25 and F27), whereas others exhibited an AMPK inhibition (F6, F8, F16, F17 and F28) (Fig 4B).

**Fig 5:**
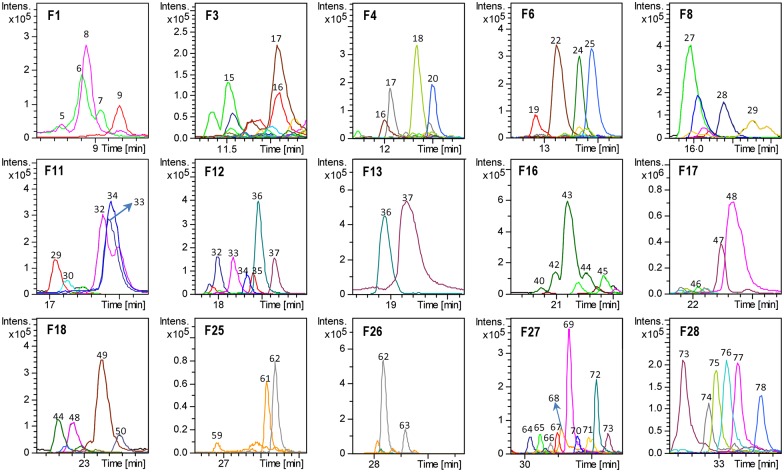
HPLC-MS characterization of the Olive-tree leaves fractions. Base peak chromatogram (BPC) of the selected fractions (F1-F28) in negative ion mode obtained by RP-HPLC-ESI-TOF/MS. The resulting peaks are identified with numbers 1–78, according to the order of elution.

Those fractions showing activation/inhibition capacity or expression level modulation of AMPK compared to the control were analyzed and fully characterized at the analytical scale by RP-HPLC-ESI-TOF/MS, as described in the Methods, to identify the responsible compounds for such an effect (Fig 5). The rest of the isolated fractions were not subjected to subsequent RP-HPLC-TOF/MS analysis.

Among the fractions showing AMPK activation (Fig 5), fraction 3 contained compounds 15 (oleoside/secologanoside isomer 4), 16 (elenolic acid glucoside/methyloleoside isomer 2), and 17 (*p*-coumaric acid glucoside), the latter being the most abundant compound. Fraction 4 contained compounds 16, 17, 18, and 20 (unknown); fraction 11 was composed of small quantities of compounds 29 (unknown), 30 (demethyloleuropein), and larger quantities of 32 (hydroxyoleuropein/hydroxyoleuroside isomer 1), 33 (glucosyl rhamnosylquercetin isomer 2), and 34 (luteolin rutinoside isomer 2). Fraction 12 contained compounds 32, 33, 34, 35 (olivil), 36 (verbascoside) as major compounds, along with 37 (luteolin glucoside isomer 1). Fraction 13 contained compounds 36, and 37; fraction 18 contained compounds 44 (oleuropein glucoside/neonuezhenide isomer 5), 48 (luteolin glucoside isomer 2), and 49 (diosmetin glucoside) as the major compounds, along with compound 50 (calceolarioside isomer 1). Fraction 25 contained compounds 59 (oleuropein/oleuroside isomer 4), 60 (unknown), and 61 (unknown). Finally, fraction 27 mostly contained compounds 69 (luteolin) and 72 (unknown) and minor amounts of compounds 64 (unknown), 65 (dimethyl hydroxy octenoyloxi secologanoside isomer 2), 66 (ligstroside isomer 2), 67 (oleuropein methyl ether), 68 (oleuropein/oleuroside isomer 5), 70 (quercetin), 71 (resinoside) and 73 (unknown).

In contrast, some other fractions showed a significant inhibition of AMPK activity (Fig 5) Fraction F6 contained compounds 22 (elenolic acid glucoside/methyloleoside isomer 3), 24 (oleuropein aglycone), and 25 (glucosyl rhamnosyl quercetin -–rutin—isomer), along with minor quantities of compound 19 (unknown). F8 contained mostly compound 27 (phenethyl primeveroside) and also significant amounts of compounds 28 (ethyl-glucopyranosyloxy-oxopropyl-cyclohexaneacetic acid) and 29 (unknown). F16 mostly contained compound 43 (oleuropein glucoside isomer 4) and minor quantities of compounds 40, 42, 44 (oleuropein glucoside/nuezhenide isomers 2, 3, and 5, respectively), and 45 (diosmetin rhamnoside glucoside—diosmin—isomer 1). F17 contained compounds 47 (apigenin glucoside) and 48 (luteolin glucoside isomer 2) and minor quantities of compound 46 (diosmetin rhamnoside glucoside—diosmin—isomer 2). Finally, F28, which drastically decreased the level of AMPK activation through phosphorylation by 30% (p<0.001), contained compounds 73 (unknown), 74 (trihydroxystearic acid), 75 (apigenin), 76 (trihydroxy-octadecenoic acid), 77 (unknown), and 78 (dihydroxyhexadecanoic acid).

Among those fractions showing a potent AMPK activation capacity through phosphorylation, a distinct behavior was observed for the total AMPK expression levels. The total AMPK levels were not significantly altered by fractions 3, and 4, were significantly increased (p<0.001) by fraction 11, and were significantly reduced (p<0.001) by fractions 12, 13, 18, 25 and 27. Most fractions with inhibitory AMPK activity showed no change in the total AMPK expression levels, i.e., fractions 6, 16, 17 and 28. However, fraction 8 exhibited decreased AMPK expression levels compared to the control.

### Molecular docking of the identified compounds against AMPK binding sites

**Fig 6:**
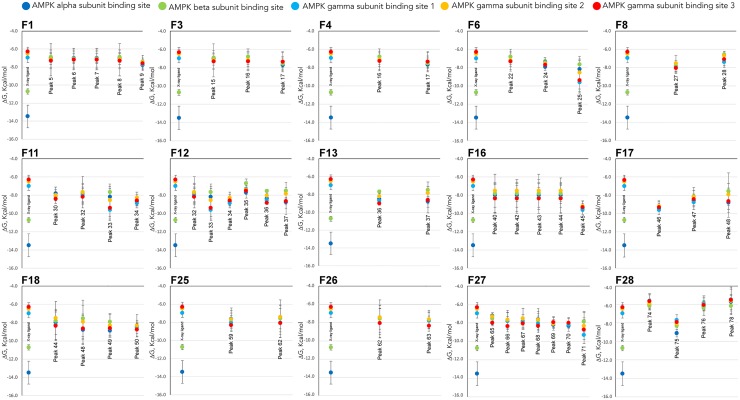
Comparison of the free energy variation (ΔG) of the Olive-tree leaves phenolic compounds and ligand against the binding sites of the three AMPK subunits obtained by molecular docking. Comparison of the free energy variation (ΔG) for all the identified compounds (Table 1 and S2 Fig) contained in those fractions showing activation/inhibition capacity of AMPK (Figs 4 and 5), based on molecular docking analysis against all known binding sites of AMPK (S3 Fig).

The aim of molecular docking is to predict the structure of the ligand-receptor complex using computational methods. In the current study, all known x-ray structures of AMPK alpha, beta and gamma have been docked with the identified compounds of each fraction (see S3 Fig). The Gibbs free energy variation (ΔG) is represented in Fig 6. All solved structures for the different subunits of AMPK have different co-crystallized ligands, which allows us to define the binding sites on which to perform the molecular docking (see S3 Fig). Additionally, we have performed molecular docking with the x-ray crystallographic ligands over its binding sites as a control of the best ΔG in each binding site. All three x-ray ligands of the AMPK gamma subunit present a ΔG ≈ – 6.5 ± 0.5 Kcal/mol when docked over its respective binding sites (PDB: 2UV4, 2UV5, 2UV6, 2UV7, 4CFE, 4CFF, 4RER, 4REW, and 4ZHX). As we can see in Fig 6, when the compounds contained in the Olive-tree leaves fractions bearing biological activity over AMPK (Fig 5) were docked to these three sites of the gamma subunit, the ΔG was less than or equal to the corresponding value for the crystallographic ligands. These results may indicate that these Olive-tree leaves compounds might act as potential ligands for the AMPK gamma subunit.

In contrast, when the same compounds were docked against the AMPK alpha subunit binding site or the AMPK beta subunit regulatory binding site, the ΔG for the Olive-tree leaves compounds exhibited higher values than those shown by the respective x-ray ligands of these binding sites. Only a few compounds showed ΔG values that were relatively close to those observed for the x-ray ligands, i.e., lower than– 8.0 ± 0.5 Kcal/mol. These included compounds 34, 36, 37, 45, 46, 47, 48, 49, 69, 70, 71 and 75 for the AMPK alpha subunit and compounds 34, 45, 46, 47, 50, 69, 70 and 75 for the AMPK beta subunit regulatory binding site.

## Discussion

Plant polyphenols are the most intensively studied natural products, as they are a recognized source of active compounds used to alleviate noncommunicable diseases such as obesity [16,27]. Plant derived polyphenols such as epigallocatechin gallate from green tea, resveratrol from grapes, curcumin from turmeric, quercetin from onion, phenylpropanoids from *Lippia citriodora*, or anthocyanins/flavonols from *Hibiscus sabdariffa* [4,16,17,24,28] have demonstrated either the inhibition of adipogenesis or the attenuation of intracellular lipid accumulation in 3T3-L1 adipocytes, a well characterized model of *in vitro* adipogenesis that becomes hypertrophic and insulin resistant when induced with high-glucose conditions [16,23,24]. Nevertheless, to date, there are no other reports on this ameliorative effect for Olive-tree leaves extracts. Our results suggest that Olive-tree leaves extracts have the potential to reduce lipid accumulation in hypertrophic and insulin resistant adipocytes. AMPK activation by phosphorylation at Thr-172 has also been correlated with a reduced lipid accumulation in the cytoplasm of 3T3-L1 adipocytes by several phenolic extracts [16,24,29], which is in agreement with our results of Olive-tree leaves extracts from using hypertrohic adipocytes.

In this regard, several lines of evidence have indicated a direct link between AMPK and lipid metabolism. It has been reported that AMPK exerts multiple effects that lead to the normalization of lipid metabolism in adipose tissue. It directly phosphorylates acetylCoA carboxylase (ACC), which leads to the inhibition of fatty acid synthesis and the stimulation of fatty acid uptake into mitochondria through malonylCoA decrease [15,30]. It inhibits lipolysis through the phosphorylation of hormone sensitive lipase [31]. It inhibits the synthesis of triglycerides and phospholipids via the inactivation of glycerol phosphate acyl transferase (GPAT), and it downregulates the transcription of lipogenic genes, including those encoding ACC, fatty acid synthase (FAS), and GPAT [32,33]. Although the Olive-tree leaves extract is able to reduce the intracellular lipid accumulation in 3T3-L1 hypertrophic adipocytes through AMPK-dependent mechanisms, more studies will need to be performed to decipher which downstream targets are involved.

The fractionation of the Olive-tree leaves extract through semi-preparative RP-HPLC-ESI-TOF/MS allowed us to delimit the number of possible candidate compounds responsible for the observed activation of AMPK. Specifically, the components that might contribute to this effect in a higher extent belonged to the secoiridoids (isomers of oleoside/secologanoside, elenolic acid glucoside/methyloleoside, demethyloleuropein, hydroxyoleuropein/hydroxyoleuroside, oleuropein glucoside/neonuezhenide, oleuropein/oleuroside isomers, dimethyl hydroxy octenoyloxi secologanoside, ligstroside, oleuropein methyl ether), the cinnamic acids, phenylethanoids and phenylpropanoids (*p*-coumaric acid glucoside, verbascoside, and calcelarioside isomers), the flavonoids (isomers of glucosyl rhamnosylquercetin—rutin–, luteolin rutinoside, luteolin glucoside, diosmetin glucoside, luteolin, quercetin, resinoside), and the lignans (olivil) subclassess. The fact that fractions 12 and 13 share compounds 36 (verbascoside) and 37 (luteolin glucoside isomer 1) and that the adjacent fraction (14) contained almost exclusively compound 37 and did not exhibit AMPK activation suggests that verbascoside could be the main agent responsible for AMPK activation in these fractions. However, confirmation with the pure standard should be carried out to verify this hypothesis. Moreover, elenolic acid glucoside and -coumaric glucoside, which are both present in fractions 3 and 4, diosmetin glucoside in fraction 18, unknown compounds 61 and 62 in fraction 25 and luteolin and unknown compound 72 in fraction 27, might contribute to the observed AMPK activation in these particular fractions.

It should be highlighted that fractions F11, F12 and F13, which contained verbascoside (F12 and F13), and hydroxyoleuropein, rutin and luteolin rutinoside (F11 and F12) exhibited the maximum level of AMPK activation, and this activation was as potent as that obtained with the AMPK synthetic activator AICAR. In agreement with our results, lemon verbena polyphenolic extract and its major compound verbascoside have recently exhibited the capacity to activate AMPK concomitantly to the peroxisome proliferator-activated receptor gamma (PPAR-gamma)-dependent transcriptional upregulation of adiponectin, the upregulation of PPAR-alpha mRNA expression and the downregulation of the mRNA expression of fatty acid synthase [16].

The molecular mechanism by which these Olive-tree leaves polyphenols exert their activation needs to be elucidated. On the one hand, these compounds may reach upstream AMPK targets such as LKB1 or CaMKKβ kinases, adipokines or ROS-dependent calcium channel activation [10,11,14]. Furthermore, our *in silico* study also suggests the possibility that some Olive-tree leaves polyphenols may act as direct modulators of AMPK at different binding sites. The binding of AMP to the gamma subunit causes the allosteric activation of AMPK kinase. The binding of either AMP or ADP promotes and maintains the phosphorylation of threonine 172 within the activation loop of the kinase [8]. Increased AMPK activity leads to a concomitant increase in the phosphorylation of its downstream targets. In addition to regulation by adenine nucleotides, a number of small molecules have been identified that directly activate AMPK [34]. The kinase domain of the α-subunit and the carbohydrate-binding module of the β-subunit are connected and define a new regulatory binding site (see S3 Fig, panels C and D). Molecular docking of the A-769662 compound, a small activator of AMPK kinase [35] for this regulatory site, presents a ΔG ≈ – 10.72 ± 0.30 Kcal/mol. Several compounds identified by RP-HPLC-TOF/MS show good enough ΔG values to be considered as putative AMPK ligands or regulators, i.e., -10.1 ± 0.8 < ΔG < 8.0 ± 0.2 Kcal/mol. As an example, the compounds demethyloleuropein, 10-hydroxyoleuropein, (7''S)-hydroxyoleuropein, luteolin 7-rutinoside, diosmin, quercetin, etc., satisfy this condition and could, therefore, have a regulatory effect on this binding site, although obviously with less intensity than the activator A-769662. Staurosporine is an ATP-competitive kinase inhibitor that binds to many kinases with a high affinity, though with little selectivity. This compound is co-crystalized at the ATP-binding site of the AMPK alpha subunit (PDB: 4CFE, 4CFF, 4ZHX, and 5EZV), and its molecular coupling shows a ΔG ≈ – 13.5 ± 1.3 Kcal/mol. Free energy variation of all identified compounds from the Olive-tree leaves AMPK alpha subunit have significantly higher values than staurosporine; therefore, no inhibitory effect on the AMPK catalytic subunit should be expected for these compounds. However, some of these compounds could act as fine regulators of the AMPK alpha subunit and beta subunit.

It has been reported that polyphenols such as *p*-coumaric, luteolin, and quercetin, in their aglycone form, are able to increase the phosphorylation of AMPK in 3T3-L1 cells [16,31,36,37]. Unlike previous findings, our results also suggest that some of the phenolic compounds from Olive-tree leaves exhibit AMPK activation as glycosides when added to the extracellular media. In agreement with our results, some authors have demonstrated the antiadipogenic effect of luteolin glucoside on 3T3-L1 adipocytes, which might indicate that such phenolic glucosides could either be able to go through the plasmatic membrane [15] or could be metabolized to luteolin aglycone at the intracellular level. The fact that the treatment of adipocytes or hepatocytes with PPAR activators, such as plant polyphenols [26], enhanced glucuronidation activity and UDP-glucuronosyltransferase expression suggests that the glucuronide metabolites of polyphenols may also reach intracellular targets [38]. Surprisingly, other authors have found an adipogenic effect of quercetin (10 μM) on human mesenchymal stem cells [39]. This apparent discrepancy may be attributed to the different experimental model used or even to the different adipogenic induction timing. In any case, the inhibitory capacity of quercetin on adipogenesis is well documented in murine cell and animal model [28,40]. An explanation could be that differentiation into adipocytes may be independent of mitotic clonal expansion depending on the type of cell line.

However, we should express caution when extrapolating cell model data to in vivo conditions. Most glycosylated phenolic compounds are converted into their aglycones on the cell surface of the intestinal epithelial cells and bacteria. Upon absorption, the aglycone forms are subjected to enzymatic modification, and their different metabolites, (glucuronide, sulfated, or methylated metabolites) reach their target tissues and organs [24].

Most of the fractions showing inhibitory activity on AMPK (F6, F8, F16, F17 and F28) behaved as mild inhibitors. Nevertheless, the potent inhibitory effect of fraction 28 (*p*<0.001), which contains the flavone apigenin among other compounds, should be noted. Although apigenin has been shown to have an antiadipogenic effect through AMPK activation in the adipocyte model [41], the inhibitory effect of this fraction could be due to the inhibition of AMPK reported for some saturated fatty acids also present in this fraction [42]. In relation to the potential inhibitory mechanism, it is known that the phosphorylation of AMPK is reversed by phosphatases, although the exact mechanisms that modulate this action remain poorly understood [43].

Although beyond the scope of the present study, it has been found that the overactivation of AMPK has been observed in several neurodegenerative diseases, such as Alzheimer’s disease, progressive supranuclear palsy, corticobasal degeneration, Pick’s disease, Parkinson’s disease, and others [7]. Therefore, AMPK inhibitors could also be relevant as therapeutic therapies against these types of diseases.

Some secoiridoids from extra virgin olive oil, i.e., oleuropein aglycone and decarboxymethyl oleuropein aglycone, have been demonstrated to activate AMPK, which results in the inhibition of the mammalian target of rapamycin (mTOR) in breast cancer cells [18,27]. It has been postulated that chronic diseases associated with aging, such as cancer or obesity, are driven by the overactivation of the nutrient-sensing mTOR gerogene due to the loss of responsiveness to active AMPK, a suppressor of mTOR. In this manner, some secoiridoids from the Olive-tree may act as gerosuppressors, triggering AMPK activation and exerting a transcriptomic signature that reduces mTOR-driven aging. In agreement with this assumption, our findings support the hypothesis that some secoiridoids from *Olea europaea* (oleoside/secologanoside, elenolic acid glucoside, demethyloleuropein/demethyloleuroside, oleuropein/oleuroside, oleuropein glucoside and hydroxyoleuropein/ hydroxyoleuroside) may contribute to the activation of AMPK by Olive-tree leaves extract in 3T3-L1 adipocytes, although this should be confirmed using isolated pure compounds in the cell model.

Due to the diverse nature of the natural AMPK activators, one important question arises: how do they manage to activate AMPK despite the fact that their structures are so different? A previous study that used a cell line expressing an AMP- and ADP- insensitive AMPK mutant proposed that phenolic compounds such as quercetin activate AMPK indirectly by increasing cellular AMP and ADP levels, usually by inhibiting mitochondrial ATP synthesis [44]. It has also been reported that oleuropein aglycone indirectly actives AMPK through calcium concentration increases and the subsequent activation of CaMKKβ in SH-SY5Y neuroblastoma cells [19]. Nevertheless, the direct activation of kinases, such as AMPK, by phenolic compounds at the nucleotide binding site has also been proposed [45,46], which is consistent with our results. Whether phenolic compounds exert their AMPK modulation through direct or indirect mechanisms in our cell model should be further confirmed.

Our evidences support that Olive-tree may become a source for bioactive compounds with a potential use in chronic human diseases. Nevertheless, olive-tree leaves composition show a remarkable variability due to location, climate, cultivation practices and seasonal factors. Understanding the factors that control the genetic basis of fruit and leave composition may be necessary for the harvesting and production of suitable extracts to be applied in human health [47,48].

## Conclusion

Olive-tree leaves extract decreases intracellular lipid accumulation through AMPK-dependent mechanisms in a hypertrophic and insulin resistant adipocyte model. The fractionation of the extract revealed that fractions containing compounds that belonged to the secoiridoids, cinnamic acids, phenylethanoids and phenylpropanoids, flavonoids and lignans subclasses were the best candidates to account for such effects. Our *in silico* results suggest the possibility that some of these compounds may be considered putative AMPK ligands or regulators at the three sites of the AMPK gamma subunit. However, the interaction of these compounds with the AMPK alpha subunit binding site or the AMPK beta subunit regulatory binding site is less probable, although a subtle regulation may occur. In conclusion, Olive-tree leaves compounds deserve further attention as a therapeutic aid in the management of obesity and/or its associated disturbances. Nevertheless, further research using pure isolated compounds in cell and animal models and including cell metabolic studies will be required to identify the compounds responsible for such effects.

## Supporting information

S1 Fig Proposed molecular structure of the identified compounds showed on Table 1 for selected fractions with biological activity over AMPK kinase. (DOCX)

S2 Fig Base Peak Chromatogram (BPC) made by HPLC-ESI-TOF. This BPC was obtained in negative ion mode of the olive leaf extract, with the optimized semi-preparative conditions, and the collected fractions highlighted in blue.
(DOCX)

S3 Fig AMPK binding sites. Panels A, C and E show the secondary structure of alpha, beta and gamma subunits of AMPK kinase (4CFE.pdb), respectively. Panels B, D and F represent the electrostatic potential surface with a co-crystalized ligand indicating the position of each binding sites.
(DOCX)

## Abbreviations

ADP: adenosine diphosphate
AICAR: 5-Aminoimidazole-4-carboxamide ribonucleotide
AMP: adenosine monophosphate
AMPK: AMP-activated protein kinase
BCS: bovine calf serum
BPC: Base peak chromatogram
CaMKKβ: Calcium/calmodulin-dependent protein kinase 2
DEX: Dexamethasone
DMEM: Dulbecco’s modified Eagle’s medium
FA: fatty acid
FBS: fetal bovine serum
HPC: high-precision calibration
IBMX: 3-isobutyl-1-methylxanthine
LKB1: liver kinase 1
mTOR: mammalian target of rapamycin
pAMPK: phospho AMP-activated protein kinase
PVD: polyvinyldifluoride
RP-HPLC-ESI-TOF/MS: Reversed-phase High-Performance Liquid Chromatography coupled to Electrospray Time-of-Flight Mass Spectrometry
Thr172: Threonine172
WHO: World Health Organization
