# Supplementary material for: AMPK modulatory activity of olive–tree leaves phenolic compounds: Bioassay-guided isolation on adipocyte model and in silico approach
Source: PLoS One. 2017 Mar 9;12(3):e0173074. doi: 10.1371/journal.pone.0173074 (PMC5344353; doi:10.1371/journal.pone.0173074)
Supplement: S1 Fig — (DOCX) [file pone.0173074.s001.docx]

**AMPK modulatory activity of olive**–**tree leaves phenolic compounds: bioassay-guided isolation on adipocyte model and in silico approach**

Cecilia Jiménez–Sánchez^1,2^, Mariló Olivares–Vicente^3^, Celia Rodríguez–Pérez^1,2^, María Herranz–López^3^, Jesús Lozano–Sánchez^1,2^, Antonio Segura–Carretero^1,2^, Alberto Fernández–Gutiérrez^1,2^, José Antonio Encinar^3¶^, Vicente Micol^3,4*¶^

^1^ Department of Analytical Chemistry, University of Granada. Granada, Spain.

^2^ Research and Development of Functional Food Centre (CIDAF), PTS, Granada, Spain.

^3^ Instituto de Biología Molecular y Celular (IBMC), Miguel Hernández University (UMH), Elche, Alicante, Spain

^4^ CIBER: CB12/03/30038, Fisiopatología de la Obesidad y la Nutrición, CIBERobn, Instituto de Salud Carlos III (ISCIII), Palma de Mallorca, Spain.

* Corresponding author

Email: [vmicol@umh.es](mailto:vmicol@umh.es) (VM)

^¶^ These authors share co-senior authorship.

**Supporting information**

**S1 Fig. Proposed molecular structure of the identified compounds showed on Table 1 for selected fractions with biological activity over AMPK kinase.**

Peaks 5, 8 and 15.

| 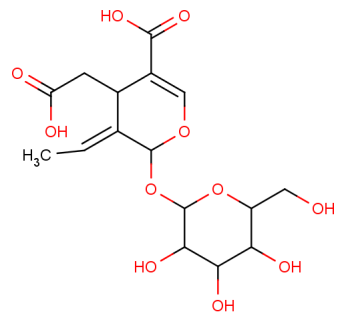 | 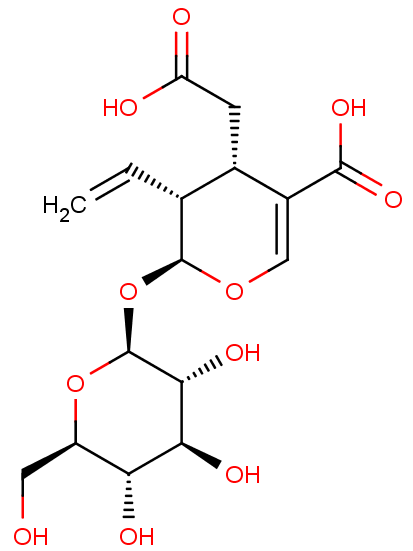 |
| --- | --- |
| 4-(carboxymethyl)-3-ethenyl-2-{[3.4.5-trihydroxy-6-(hydroxymethyl)oxan-2-yl]oxy}-3.4-dihydro-2H-pyran-5-carboxylic acid  PubChem CID: 101042548, Oleoside. | (3E)-4-(carboxymethyl)-3-ethylidene-2-{[3.4.5-trihydroxy-6-(hydroxymethyl)oxan-2-yl]oxy}-3.4-dihydro-2H-pyran-5-carboxylic acid  PubChem CID: 14136854, Secologanoside. |

Peaks 6 and 7

| 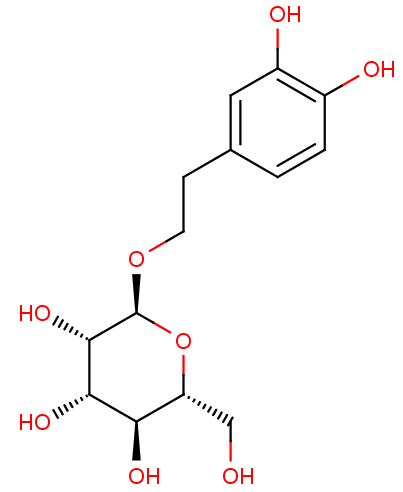 | 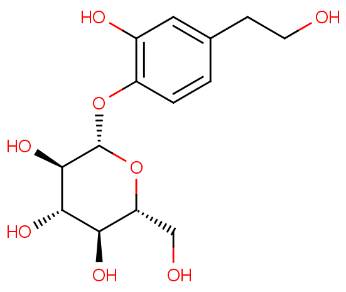 | 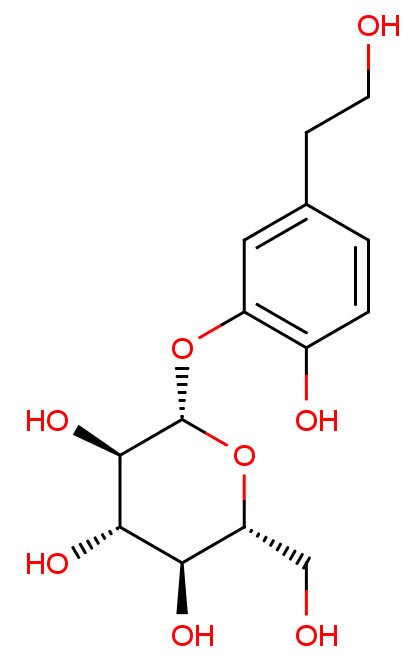 |
| --- | --- | --- |
| 2-[2-(3.4-dihydroxyphenyl)ethoxy]-6-(hydroxymethyl)oxane-3.4.5-triol  PubChem CID: 13845930, Hydroxytyrosol 1-O-glucoside. | 2-[2-hydroxy-4-(2-hydroxyethyl)phenoxy]-6-(hydroxymethyl)oxane-3.4.5-triol  PubChem CID: 6453057, Hydroxytyrosol 4-beta-D-glucoside. | 2-[2-hydroxy-5-(2-hydroxyethyl)phenoxy]-6-(hydroxymethyl)oxane-3.4.5-triol  PubChem CID: 5315870, Cimidahurinine. |

Peaks 9.

| 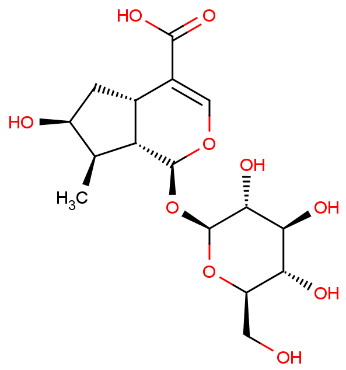 | 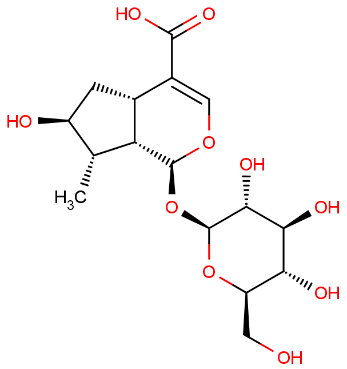 |
| --- | --- |
| 6-hydroxy-7-methyl-1-{[3.4.5-trihydroxy-6-(hydroxymethyl)oxan-2-yl]oxy}-1H.4aH.5H.6H.7H.7aH-cyclopenta[c]pyran-4-carboxylic acid  PubChem CID: 158144, 8-Epiloganic acid. | 6-hydroxy-7-methyl-1-{[3.4.5-trihydroxy-6-(hydroxymethyl)oxan-2-yl]oxy}-1H.4aH.5H.6H.7H.7aH-cyclopenta[c]pyran-4-carboxylic acid  PubChem CID: 89640, Loganic Acid. |

Peaks 16 and 22.

| 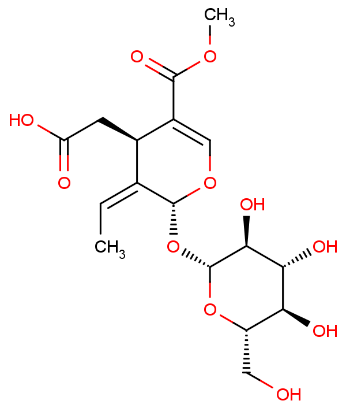 | 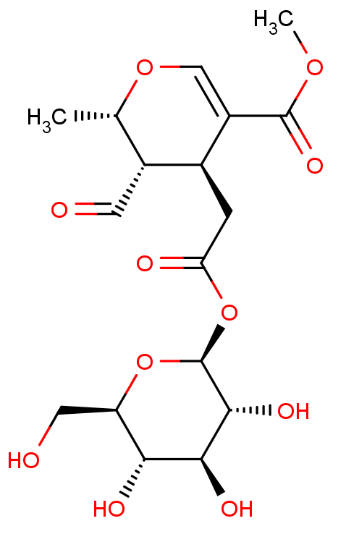 |
| --- | --- |
| methyl 3-formyl-2-methyl-4-(2-oxo-2-{[3.4.5-trihydroxy-6-(hydroxymethyl)oxan-2-yl]oxy}ethyl)-3.4-dihydro-2H-pyran-5-carboxylate  PubChem CID: 24121278, oleoside 11-methyl ester. | 2-[(3Z)-3-ethylidene-5-(methoxycarbonyl)-2-{[3.4.5-trihydroxy-6-(hydroxymethyl)oxan-2-yl]oxy}-3.4-dihydro-2H-pyran-4-yl]acetic acid |

Peak 17

| 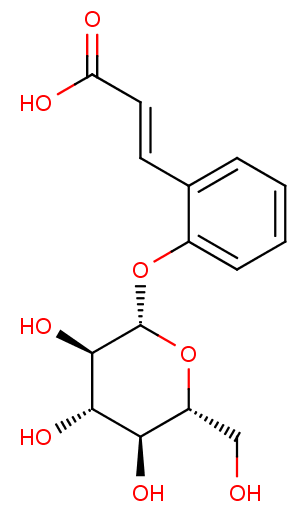 | 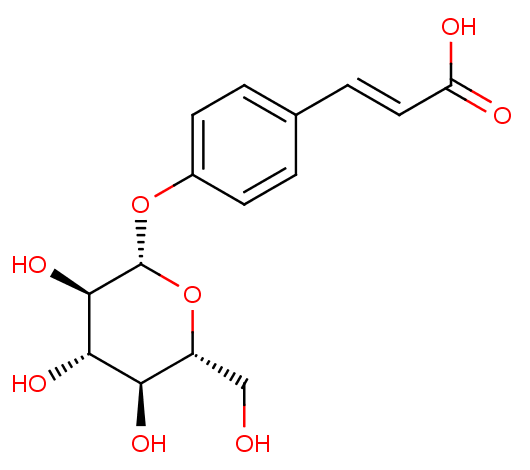 |
| --- | --- |
| (2E)-3-(2-{[3.4.5-trihydroxy-6-(hydroxymethyl)oxan-2-yl]oxy}phenyl)prop-2-enoic acid  PubChem CID: 6275271. | (2E)-3-(4-{[3.4.5-trihydroxy-6-(hydroxymethyl)oxan-2-yl]oxy}phenyl)prop-2-enoic acid  PubChem CID: 9840292. |

Peak 24

| 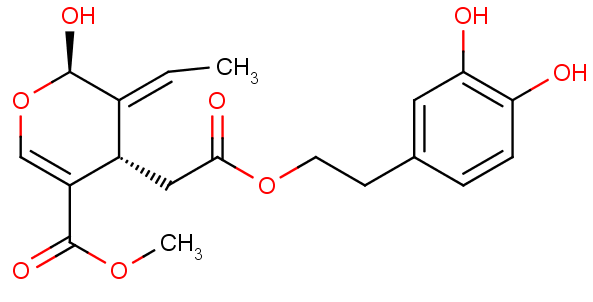 |
| --- |
| methyl (3E)-4-{2-[2-(3.4-dihydroxyphenyl)ethoxy]-2-oxoethyl}-3-ethylidene-2-hydroxy-3.4-dihydro-2H-pyran-5-carboxylate  PubChem CID: 56842347, Oleuropein Aglycone. |

Peak 25 and 33

| 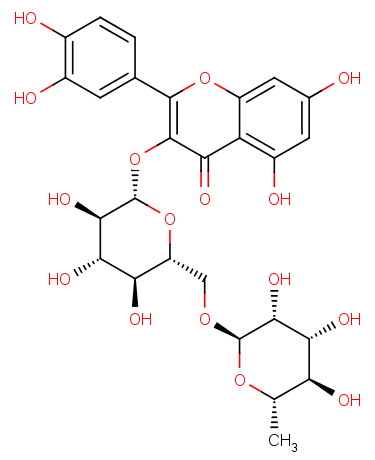 |
| --- |
| 2-(3.4-dihydroxyphenyl)-5.7-dihydroxy-3-[(3.4.5-trihydroxy-6-{[(3.4.5-trihydroxy-6-methyloxan-2-yl)oxy]methyl}oxan-2-yl)oxy]-4H-chromen-4-one  PubChem CID: 5280805, Rutin. |

Peak 27

| 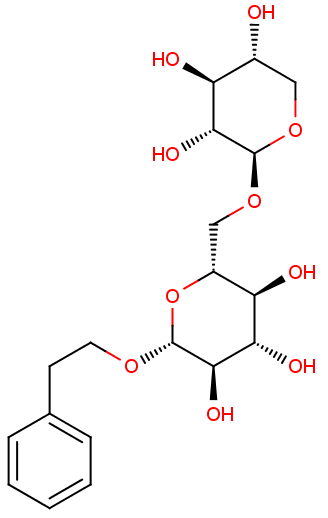 | 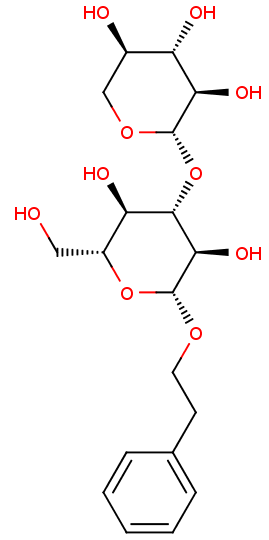 |
| --- | --- |
| 2-(2-phenylethoxy)-6-{[(3.4.5-trihydroxyoxan-2-yl)oxy]methyl}oxane-3.4.5-triol  PubChem CID: 131129, 2-Phenylethyl beta-primeveroside. | 2-{[3.5-dihydroxy-2-(hydroxymethyl)-6-(2-phenylethoxy)oxan-4-yl]oxy}oxane-3.4.5-triol  PubChem CID: 100930979. |

Peak 28

| 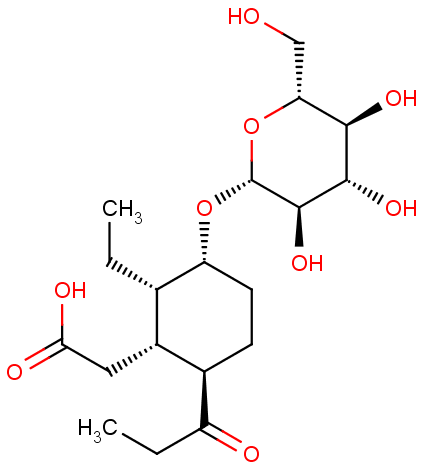 |
| --- |
| 2-(2-ethyl-6-propanoyl-3-{[3.4.5-trihydroxy-6-(hydroxymethyl)oxan-2-yl]oxy}cyclohexyl)acetic acid |

Peak 30

| 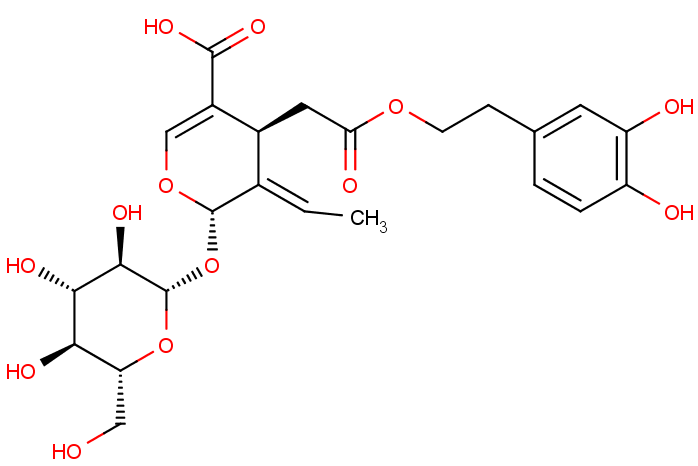 |
| --- |
| (3E)-4-{2-[2-(3.4-dihydroxyphenyl)ethoxy]-2-oxoethyl}-3-ethylidene-2-{[3.4.5-trihydroxy-6-(hydroxymethyl)oxan-2-yl]oxy}-3.4-dihydro-2H-pyran-5-carboxylic acid  PubChem CID: 6450302, Demethyloleuropein. |

Peaks 32 and 39

| 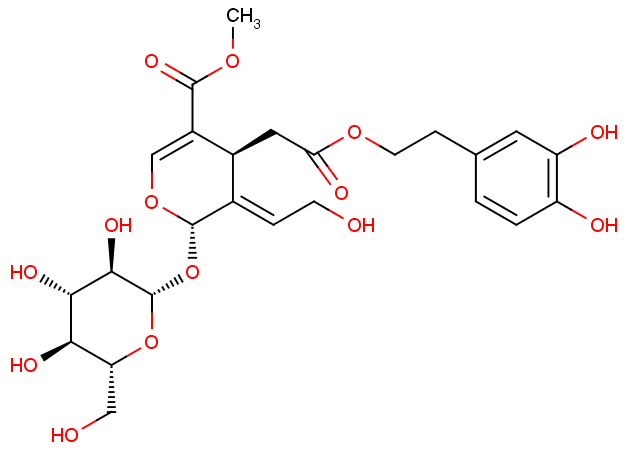 | 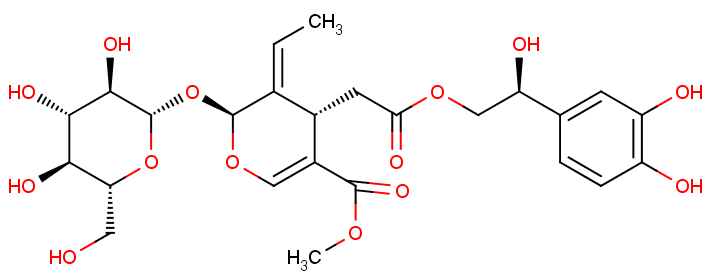 |
| --- | --- |
| methyl (3E)-4-{2-[2-(3.4-dihydroxyphenyl)ethoxy]-2-oxoethyl}-3-(2-hydroxyethylidene)-2-{[3.4.5-trihydroxy-6-(hydroxymethyl)oxan-2-yl]oxy}-3.4-dihydro-2H-pyran-5-carboxylate  PubChem CID: 6440747, 10-Hydroxyoleuropein. | methyl (3E)-4-{2-[2-(3.4-dihydroxyphenyl)-2-hydroxyethoxy]-2-oxoethyl}-3-ethylidene-2-{[3.4.5-trihydroxy-6-(hydroxymethyl)oxan-2-yl]oxy}-3.4-dihydro-2H-pyran-5-carboxylate  PubChem CID: 102461563, (7''S)-Hydroxyoleuropein. |
| 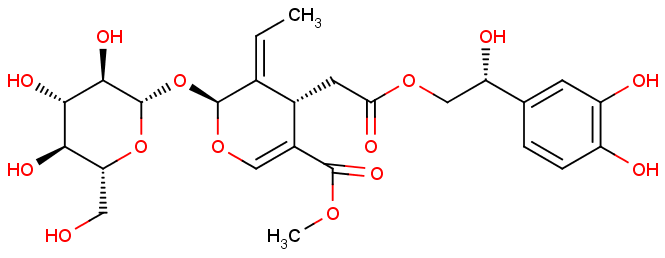 |  |
| methyl (3E)-4-{2-[2-(3.4-dihydroxyphenyl)-2-hydroxyethoxy]-2-oxoethyl}-3-ethylidene-2-{[3.4.5-trihydroxy-6-(hydroxymethyl)oxan-2-yl]oxy}-3.4-dihydro-2H-pyran-5-carboxylate  PubChem CID: 102461562, (2''R)-2''-Hydroxyoleuropein. |  |

Peak 34

| 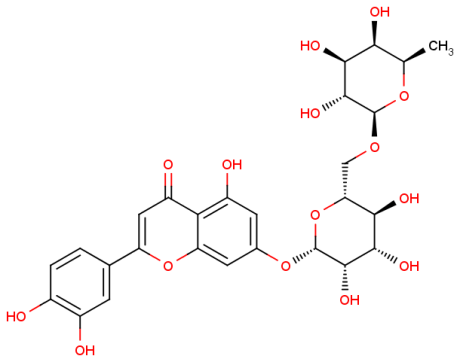 | 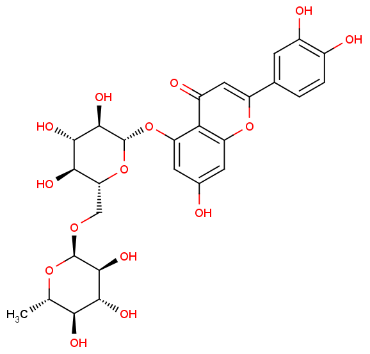 | 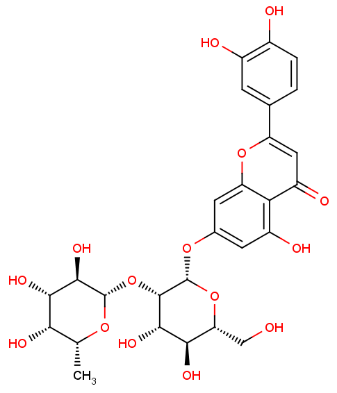 | |
| --- | --- | --- | --- |
| 2-(3.4-dihydroxyphenyl)-5-hydroxy-7-[(3.4.5-trihydroxy-6-{[(3.4.5-trihydroxy-6-methyloxan-2-yl)oxy]methyl}oxan-2-yl)oxy]-4H-chromen-4-one  PubChem CID: 44258082, Luteolin 7-rutinoside. | 2-(3.4-dihydroxyphenyl)-7-hydroxy-5-[(3.4.5-trihydroxy-6-{[(3.4.5-trihydroxy-6-methyloxan-2-yl)oxy]methyl}oxan-2-yl)oxy]-4H-chromen-4-one  PubChem CID: 44258131, Luteolin 5-O-rutinoside. | 7-{[4.5-dihydroxy-6-(hydroxymethyl)-3-[(3.4.5-trihydroxy-6-methyloxan-2-yl)oxy]oxan-2-yl]oxy}-2-(3.4-dihydroxyphenyl)-5-hydroxy-4H-chromen-4-one  PubChem CID: 44258083, Luteolin 7-neohesperidoside. | |
| 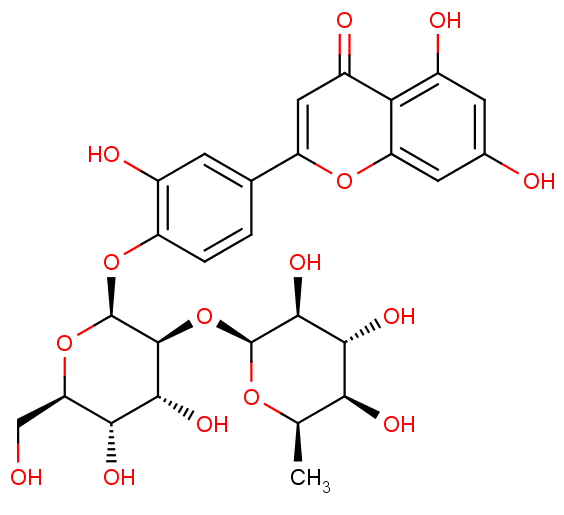 | 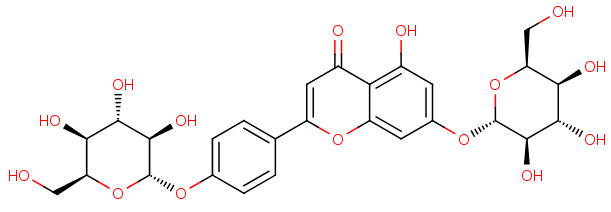 | |  |
| 2-(4-{[4.5-dihydroxy-6-(hydroxymethyl)-3-[(3.4.5-trihydroxy-6-methyloxan-2-yl)oxy]oxan-2-yl]oxy}-3-hydroxyphenyl)-5.7-dihydroxy-4H-chromen-4-one  PubChem CID: 44258098, Luteolin 4'-neohesperidoside. | 5-hydroxy-7-{[3.4.5-trihydroxy-6-(hydroxymethyl)oxan-2-yl]oxy}-2-(4-{[3.4.5-trihydroxy-6-(hydroxymethyl)oxan-2-yl]oxy}phenyl)-4H-chromen-4-one  PubChem CID: 44257819. | |  |

Peak 35

| 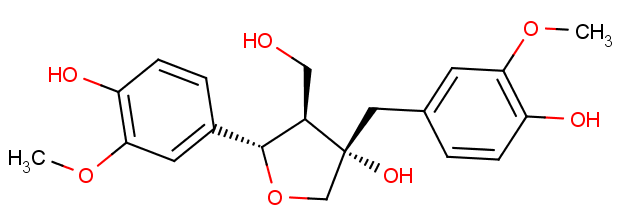 |
| --- |
| 5-(4-hydroxy-3-methoxyphenyl)-3-[(4-hydroxy-3-methoxyphenyl)methyl]-4-(hydroxymethyl)oxolan-3-ol  PubChem CID: 5273570, Olivil. |

Peak 36

| 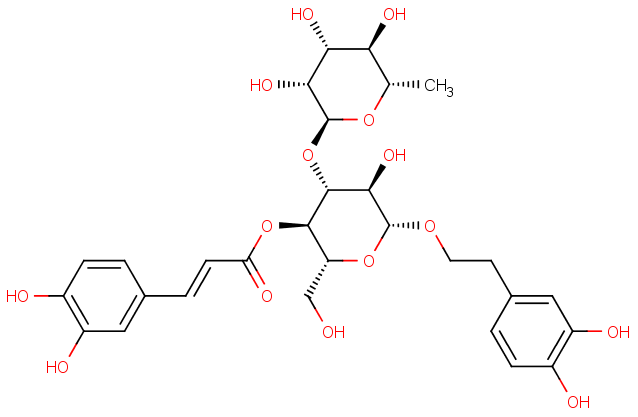 |
| --- |
| 6-[2-(3.4-dihydroxyphenyl)ethoxy]-5-hydroxy-2-(hydroxymethyl)-4-[(3.4.5-trihydroxy-6-methyloxan-2-yl)oxy]oxan-3-yl (2E)-3-(3.4-dihydroxyphenyl)prop-2-enoate  PubChem CID: 5281800, Acteoside. |

Peaks 37, 48 and 55

| 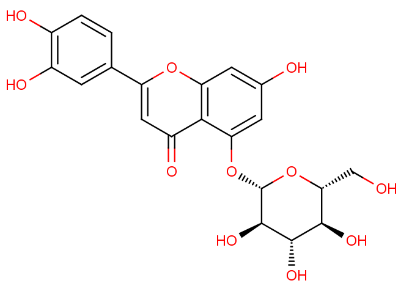 | 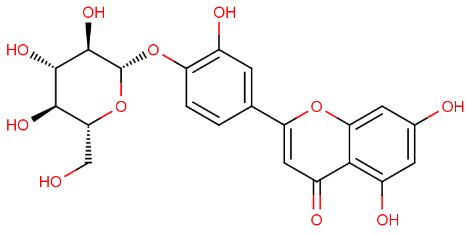 | | 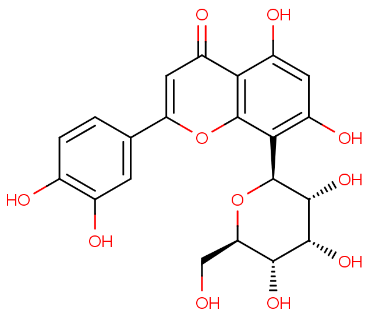 | |
| --- | --- | --- | --- | --- |
| 2-(3.4-dihydroxyphenyl)-7-hydroxy-5-{[3.4.5-trihydroxy-6-(hydroxymethyl)oxan-2-yl]oxy}-4H-chromen-4-one  PubChem CID: 5317471, Luteolin-5-O-b-D-glucopyranoside. | 5.7-dihydroxy-2-(3-hydroxy-4-{[3.4.5-trihydroxy-6-(hydroxymethyl)oxan-2-yl]oxy}phenyl)-4H-chromen-4-one  PubChem CID: 5319116, Luteolin 4'-O-glucoside. | | 2-(3.4-dihydroxyphenyl)-5.7-dihydroxy-8-[3.4.5-trihydroxy-6-(hydroxymethyl)oxan-2-yl]-4H-chromen-4-one  PubChem CID: 44257907, Luteolin 8-C-beta-D-glucopyranoside. | |
| 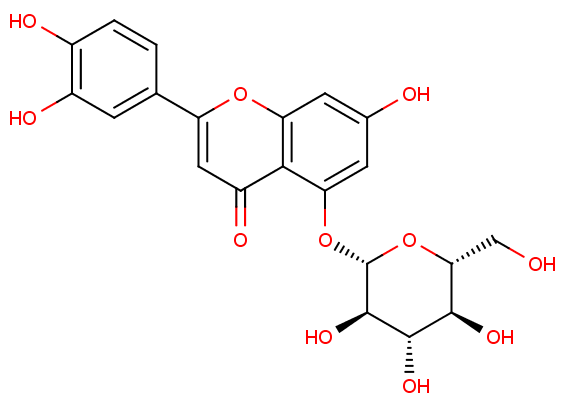 | | 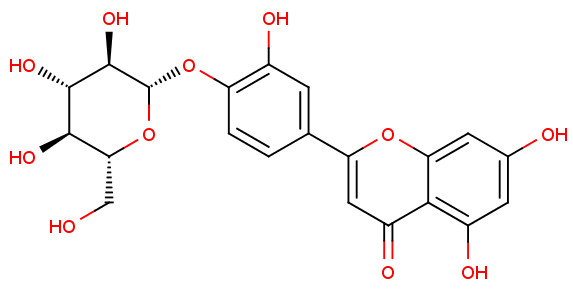 | |  |
| 2-(3.4-dihydroxyphenyl)-7-hydroxy-5-{[3.4.5-trihydroxy-6-(hydroxymethyl)oxan-2-yl]oxy}-4H-chromen-4-one  PubChem CID: 5317471, Luteolin-5-O-b-D-glucopyranoside. | | 5.7-dihydroxy-2-(3-hydroxy-4-{[3.4.5-trihydroxy-6-(hydroxymethyl)oxan-2-yl]oxy}phenyl)-4H-chromen-4-one  PubChem CID: 5319116, Luteolin 4'-O-glucoside. | |  |
| 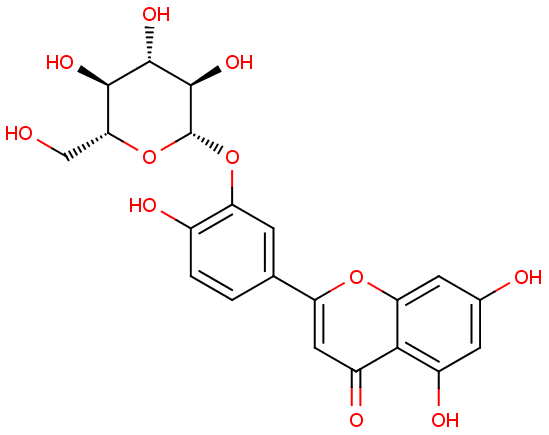 | | 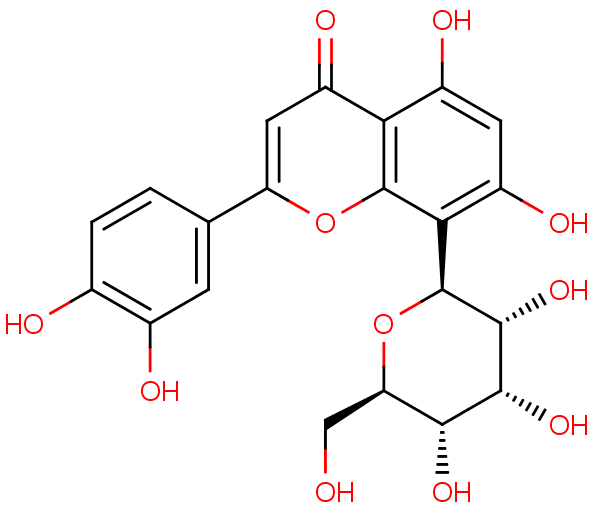 | |  |
| 5.7-dihydroxy-2-(4-hydroxy-3-{[3.4.5-trihydroxy-6-(hydroxymethyl)oxan-2-yl]oxy}phenyl)-4H-chromen-4-one  PubChem CID: 12309350, Luteolin 3'-glucoside. | | 2-(3.4-dihydroxyphenyl)-5.7-dihydroxy-8-[3.4.5-trihydroxy-6-(hydroxymethyl)oxan-2-yl]-4H-chromen-4-one  PubChem CID: 44257907, Luteolin 8-C-beta-D-glucopyranoside. | |  |

Peaks 40, 42, 43 and 44

| 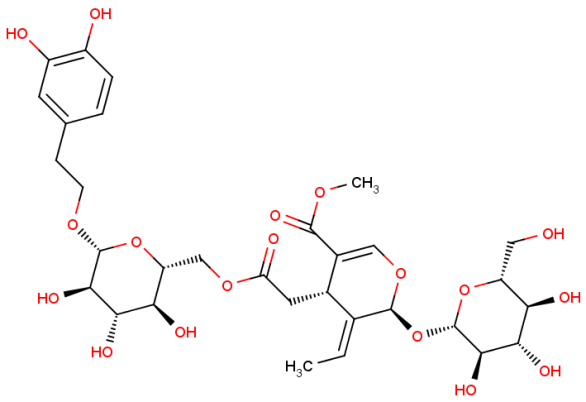 | 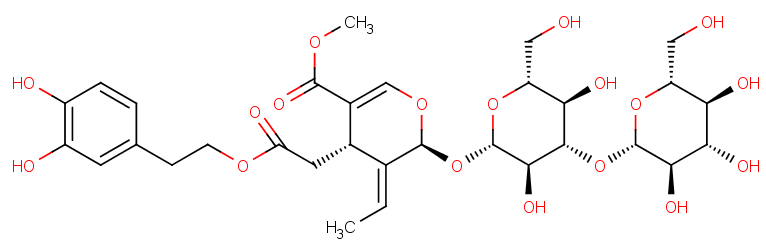 |
| --- | --- |
| methyl (3E)-4-[2-({6-[2-(3.4-dihydroxyphenyl)ethoxy]-3.4.5-trihydroxyoxan-2-yl}methoxy)-2-oxoethyl]-3-ethylidene-2-{[3.4.5-trihydroxy-6-(hydroxymethyl)oxan-2-yl]oxy}-3.4-dihydro-2H-pyran-5-carboxylate  PubChem CID: 101720830, Neonuezhenide. | methyl (3E)-2-{[3.5-dihydroxy-6-(hydroxymethyl)-4-{[3.4.5-trihydroxy-6-(hydroxymethyl)oxan-2-yl]oxy}oxan-2-yl]oxy}-4-{2-[2-(3.4-dihydroxyphenyl)ethoxy]-2-oxoethyl}-3-ethylidene-3.4-dihydro-2H-pyran-5-carboxylate  PubChem CID: 102031346, Oleuropein 3'-glucoside. |
| 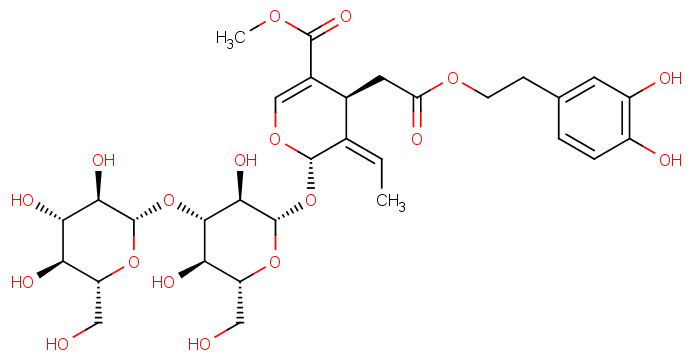 | 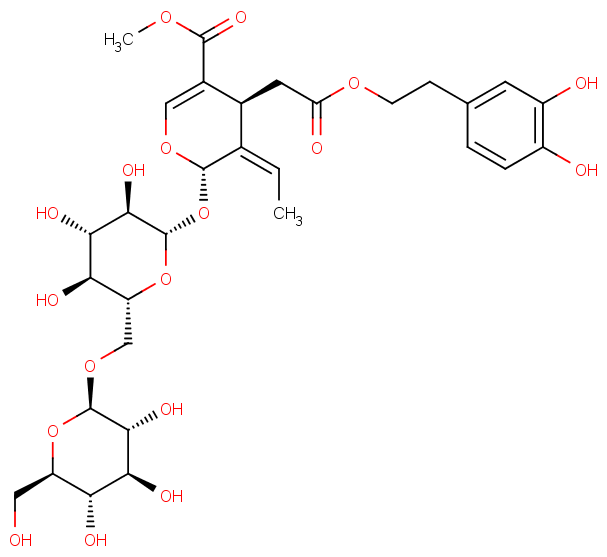 |
| methyl (3Z)-2-{[3.5-dihydroxy-6-(hydroxymethyl)-4-{[3.4.5-trihydroxy-6-(hydroxymethyl)oxan-2-yl]oxy}oxan-2-yl]oxy}-4-{2-[2-(3.4-dihydroxyphenyl)ethoxy]-2-oxoethyl}-3-ethylidene-3.4-dihydro-2H-pyran-5-carboxylate  PubChem CID: 101447987. | methyl (3Z)-4-{2-[2-(3.4-dihydroxyphenyl)ethoxy]-2-oxoethyl}-3-ethylidene-2-{[3.4.5-trihydroxy-6-({[3.4.5-trihydroxy-6-(hydroxymethyl)oxan-2-yl]oxy}methyl)oxan-2-yl]oxy}-3.4-dihydro-2H-pyran-5-carboxylate  PubChem CID: 102078602, 6'-O-beta-D-Glucopyranosyloleuropein. |

Peaks 45 and 46

| 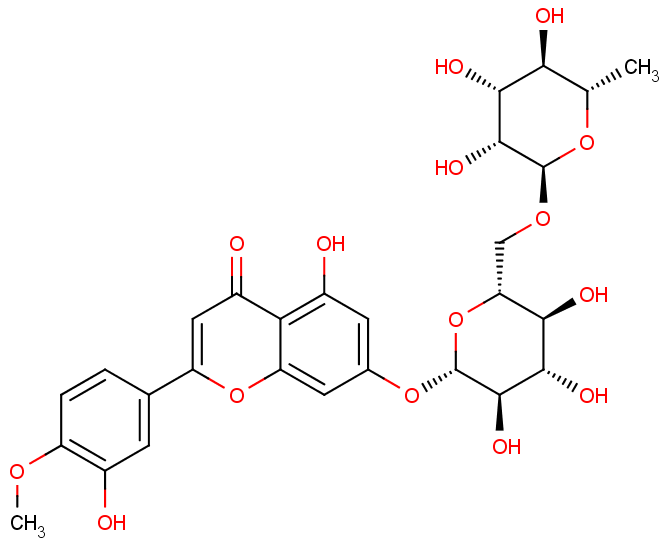 |
| --- |
| 5-hydroxy-2-(3-hydroxy-4-methoxyphenyl)-7-[(3.4.5-trihydroxy-6-{[(3.4.5-trihydroxy-6-methyloxan-2-yl)oxy]methyl}oxan-2-yl)oxy]-4H-chromen-4-one  PubChem CID: 5281613, Diosmin. |

Peak 47

| 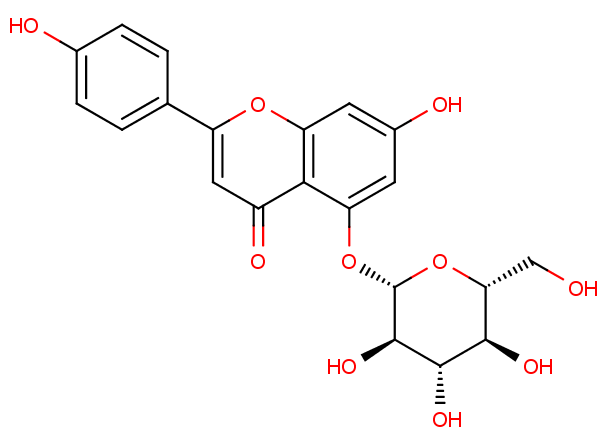 | 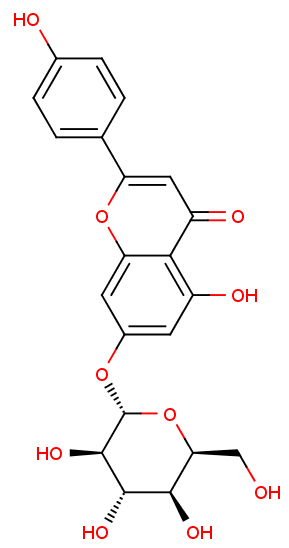 | 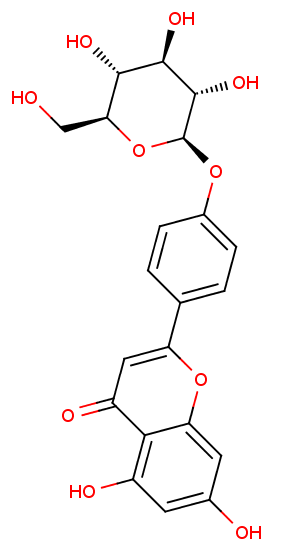 |
| --- | --- | --- |
| 7-hydroxy-2-(4-hydroxyphenyl)-5-{[3.4.5-trihydroxy-6-(hydroxymethyl)oxan-2-yl]oxy}-4H-chromen-4-one  PubChem CID: 14730806. | 5-hydroxy-2-(4-hydroxyphenyl)-7-{[3.4.5-trihydroxy-6-(hydroxymethyl)oxan-2-yl]oxy}-4H-chromen-4-one  PubChem CID: 44257792. | 5.7-dihydroxy-2-(4-{[3.4.5-trihydroxy-6-(hydroxymethyl)oxan-2-yl]oxy}phenyl)-4H-chromen-4-one  PubChem CID: 101135001. |

Peak 49

| 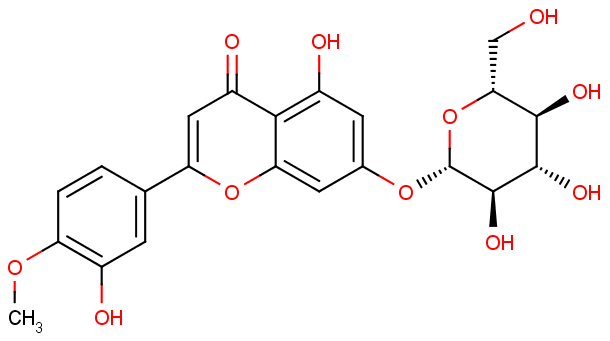 | 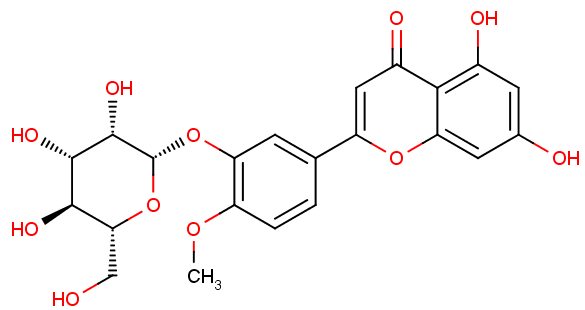 |
| --- | --- |
| 5-hydroxy-2-(3-hydroxy-4-methoxyphenyl)-7-{[3.4.5-trihydroxy-6-(hydroxymethyl)oxan-2-yl]oxy}-4H-chromen-4-one  PubChem CID: 11016019, Diosmetol 7-glucoside. | 5.7-dihydroxy-2-(4-methoxy-3-{[3.4.5-trihydroxy-6-(hydroxymethyl)oxan-2-yl]oxy}phenyl)-4H-chromen-4-one  PubChem CID: 44258229, Diosmetin 3'-glucoside. |

Peak 50

| 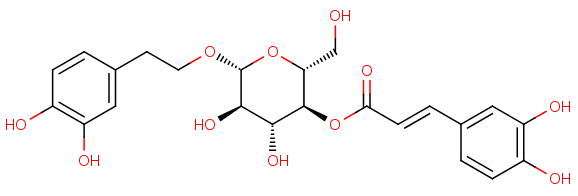 | 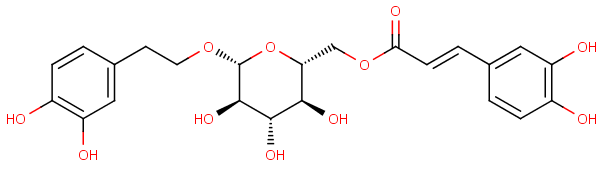 |
| --- | --- |
| 6-[2-(3.4-dihydroxyphenyl)ethoxy]-4.5-dihydroxy-2-(hydroxymethyl)oxan-3-yl (2E)-3-(3.4-dihydroxyphenyl)prop-2-enoate  PubChem CID: 5273566, Calceolarioside A. | {6-[2-(3.4-dihydroxyphenyl)ethoxy]-3.4.5-trihydroxyoxan-2-yl}methyl (2E)-3-(3.4-dihydroxyphenyl)prop-2-enoate  PubChem CID: 5273567, Calceolarioside B. |

Peaks 59 and 68

| 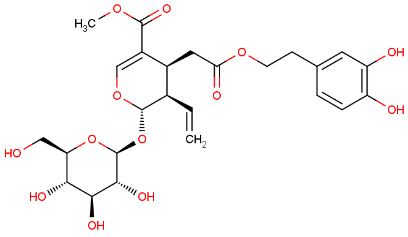 | 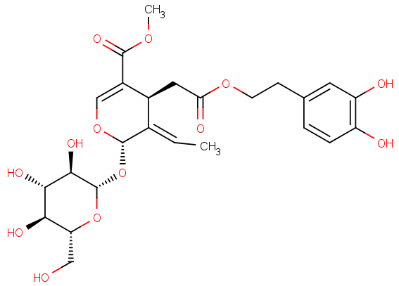 | 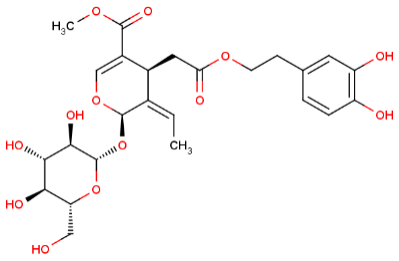 |
| --- | --- | --- |
| methyl 4-{2-[2-(3.4-dihydroxyphenyl)ethoxy]-2-oxoethyl}-3-ethenyl-2-{[3.4.5-trihydroxy-6-(hydroxymethyl)oxan-2-yl]oxy}-3.4-dihydro-2H-pyran-5-carboxylate  PubChem CID: 102016333, Oleuroside. | methyl (3E)-4-{2-[2-(3.4-dihydroxyphenyl)ethoxy]-2-oxoethyl}-3-ethylidene-2-{[3.4.5-trihydroxy-6-(hydroxymethyl)oxan-2-yl]oxy}-3.4-dihydro-2H-pyran-5-carboxylate  PubChem CID: 5281544, Oleuropein. | methyl (3Z)-4-{2-[2-(3.4-dihydroxyphenyl)ethoxy]-2-oxoethyl}-3-ethylidene-2-{[3.4.5-trihydroxy-6-(hydroxymethyl)oxan-2-yl]oxy}-3.4-dihydro-2H-pyran-5-carboxylate  PubChem CID: 53297357, Oleuropein. |

Peak 62

| 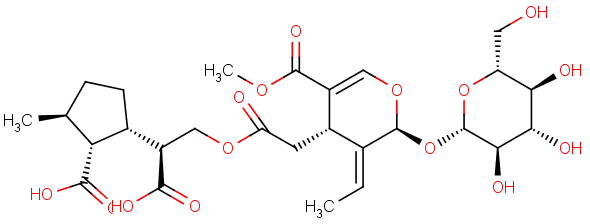 | 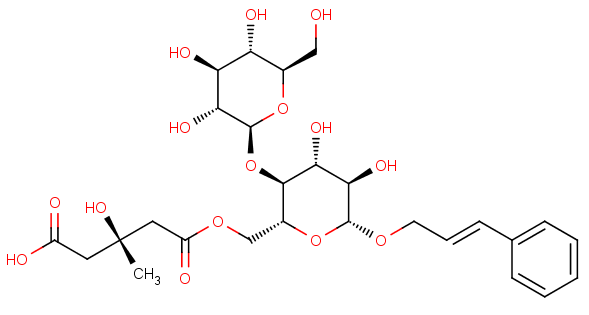 |
| --- | --- |
| 2-[1-carboxy-2-({2-[(3E)-3-ethylidene-5-(methoxycarbonyl)-2-{[3.4.5-trihydroxy-6-(hydroxymethyl)oxan-2-yl]oxy}-3.4-dihydro-2H-pyran-4-yl]acetyl}oxy)ethyl]-5-methylcyclopentane-1-carboxylic acid  PubChem CID: 102461565, Frameroside. | 5-[(4.5-dihydroxy-6-{[(2E)-3-phenylprop-2-en-1-yl]oxy}-3-{[3.4.5-trihydroxy-6-(hydroxymethyl)oxan-2-yl]oxy}oxan-2-yl)methoxy]-3-hydroxy-3-methyl-5-oxopentanoic acid  PubChem CID: 44521607, Piperchabaoside B. |
| 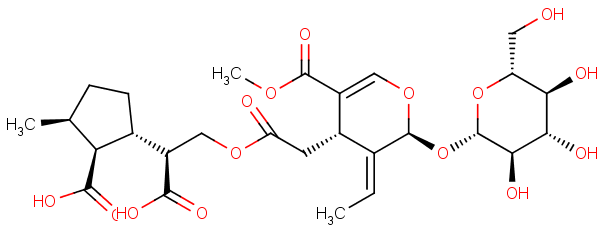 | 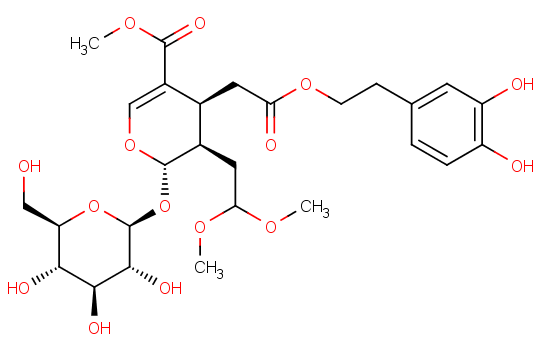 |
| 2-[1-carboxy-2-({2-[(3E)-3-ethylidene-5-(methoxycarbonyl)-2-{[3.4.5-trihydroxy-6-(hydroxymethyl)oxan-2-yl]oxy}-3.4-dihydro-2H-pyran-4-yl]acetyl}oxy)ethyl]-5-methylcyclopentane-1-carboxylic acid  PubChem CID: 11968448, 2''-epi-Frameroside. | methyl 4-{2-[2-(3.4-dihydroxyphenyl)ethoxy]-2-oxoethyl}-3-(2.2-dimethoxyethyl)-2-{[3.4.5-trihydroxy-6-(hydroxymethyl)oxan-2-yl]oxy}-3.4-dihydro-2H-pyran-5-carboxylate  PubChem CID: 44521607, Piperchabaoside B. |

Peaks 63 and 66

| 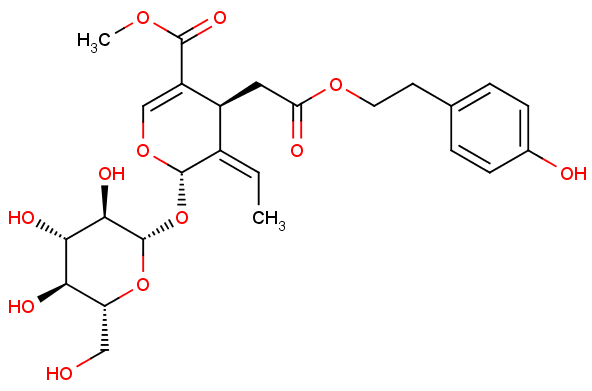 | 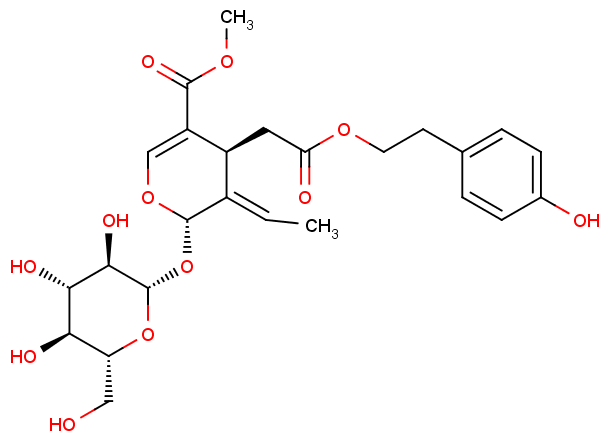 |
| --- | --- |
| methyl (3Z)-3-ethylidene-4-{2-[2-(4-hydroxyphenyl)ethoxy]-2-oxoethyl}-2-{[3.4.5-trihydroxy-6-(hydroxymethyl)oxan-2-yl]oxy}-3.4-dihydro-2H-pyran-5-carboxylate  PubChem CID: 10392063, (8Z)-Ligstroside. | methyl (3E)-3-ethylidene-4-{2-[2-(4-hydroxyphenyl)ethoxy]-2-oxoethyl}-2-{[3.4.5-trihydroxy-6-(hydroxymethyl)oxan-2-yl]oxy}-3.4-dihydro-2H-pyran-5-carboxylate  PubChem CID: 14136859, Ligstroside. |

Peak 65

| 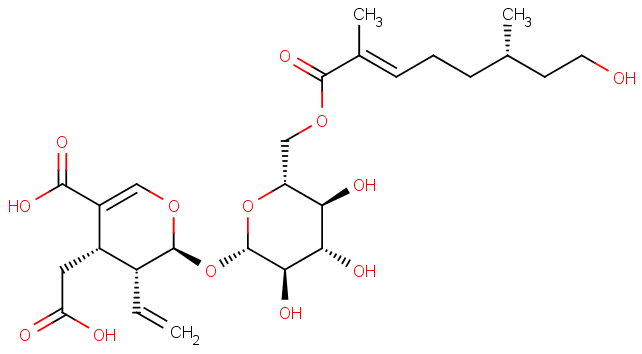 |
| --- |
| 4-(carboxymethyl)-3-ethenyl-2-{[3.4.5-trihydroxy-6-({[(2E)-8-hydroxy-2.6-dimethyloct-2-enoyl]oxy}methyl)oxan-2-yl]oxy}-3.4-dihydro-2H-pyran-5-carboxylic acid  PubChem CID: 101406919. |

Peak 67

| 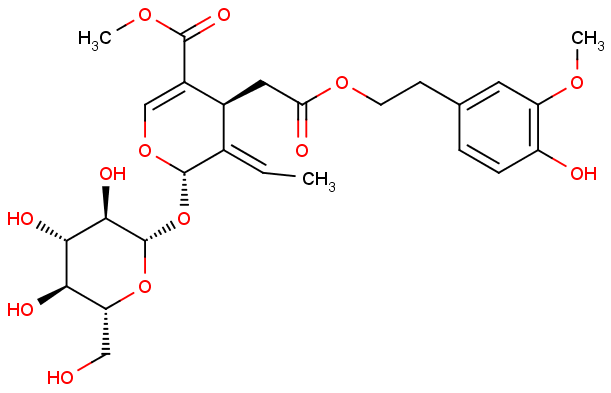 |
| --- |
| methyl (3E)-3-ethylidene-4-{2-[2-(4-hydroxy-3-methoxyphenyl)ethoxy]-2-oxoethyl}-2-{[3.4.5-trihydroxy-6-(hydroxymethyl)oxan-2-yl]oxy}-3.4-dihydro-2H-pyran-5-carboxylate  PubChem CID: 102047330. |

Peak 69

| 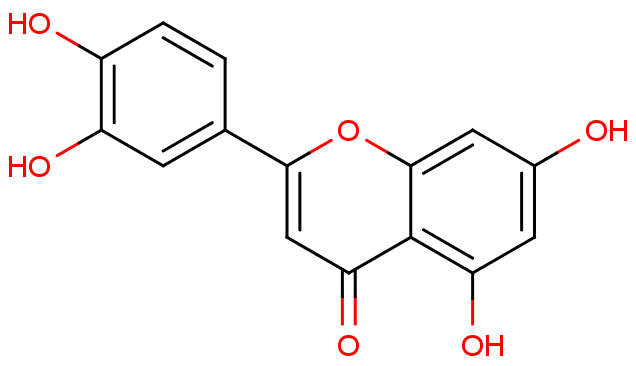 |
| --- |
| 2-(3.4-dihydroxyphenyl)-5.7-dihydroxy-4H-chromen-4-one  PubChem CID: 5280445, Luteolin. |

Peak 70

| 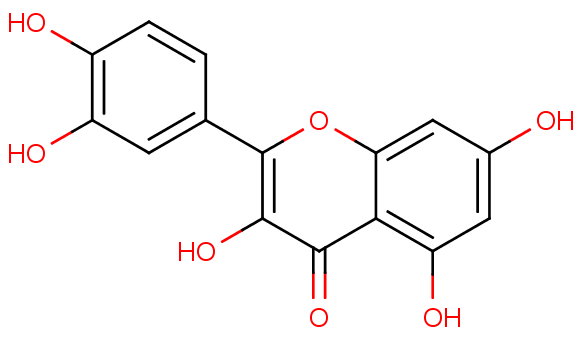 |
| --- |
| 2-(3.4-dihydroxyphenyl)-3.5.7-trihydroxy-4H-chromen-4-one  PubChem CID: 5280343, Quercetin. |

Peak 71

| 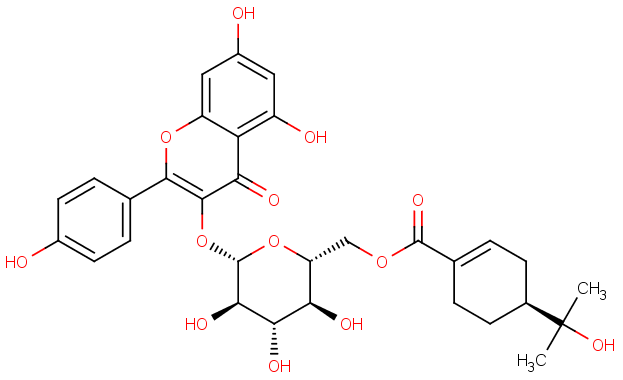 | 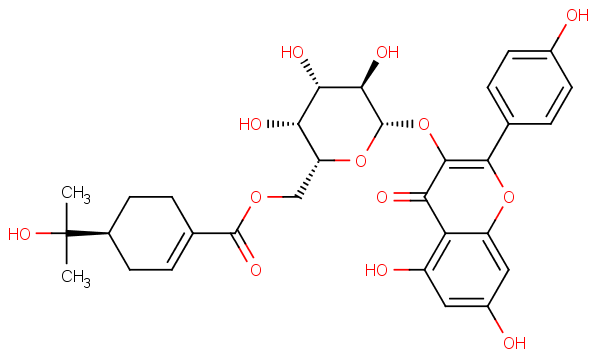 |
| --- | --- |
| (6-{[5.7-dihydroxy-2-(4-hydroxyphenyl)-4-oxo-4H-chromen-3-yl]oxy}-3.4.5-trihydroxyoxan-2-yl)methyl 4-(2-hydroxypropan-2-yl)cyclohex-1-ene-1-carboxylate  PubChem CID: 15172373, Resinoside A. | (6-{[5.7-dihydroxy-2-(4-hydroxyphenyl)-4-oxo-4H-chromen-3-yl]oxy}-3.4.5-trihydroxyoxan-2-yl)methyl 4-(2-hydroxypropan-2-yl)cyclohex-1-ene-1-carboxylate  PubChem CID: 101618995, Resinoside B. |

Peak 74

| 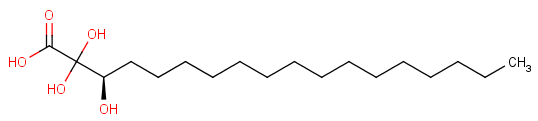 | 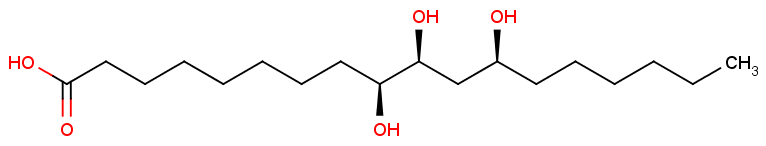 |
| --- | --- |
| 2.2.3-trihydroxyoctadecanoic acid  PubChem CID: 147011, 2,2,3-trihydroxyoctadecanoic acid. | 9.10.12-trihydroxyoctadecanoic acid  PubChem CID: 4252742, 9,10,12-trihydroxyoctadecanoic Acid. |

Peak 75

| 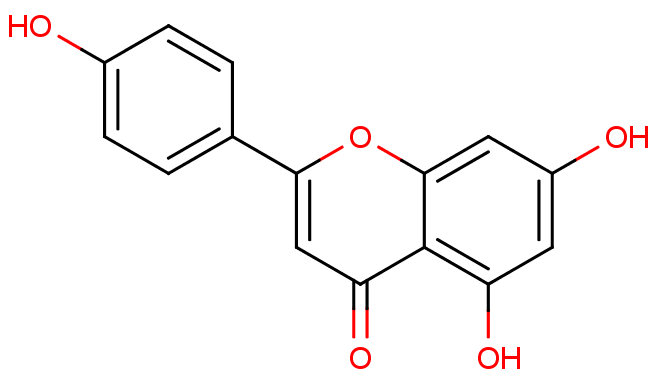 |
| --- |
| 5.7-dihydroxy-2-(4-hydroxyphenyl)-4H-chromen-4-one  PubChem CID: 5280443. |

Peak 76

| 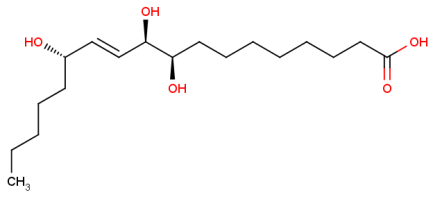 | 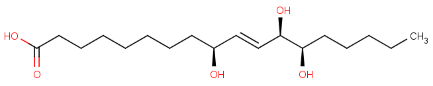 | 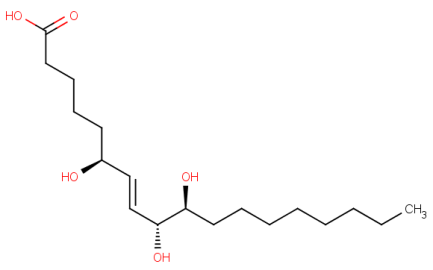 |
| --- | --- | --- |
| (11E)-9.10.13-trihydroxyoctadec-11-enoic acid  PubChem CID: 5282965. | (10E)-9.12.13-trihydroxyoctadec-10-enoic acid  PubChem CID: 5282966. | (7E)-6.9.10-trihydroxyoctadec-7-enoic acid  PubChem CID: 53248432. |

Peak 78

| 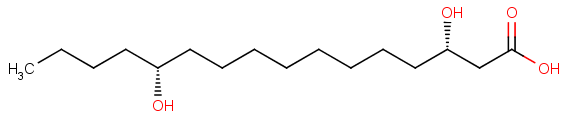 | 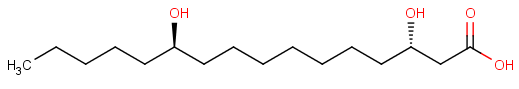 |
| --- | --- |
| 3.12-dihydroxyhexadecanoic acid  PubChem CID: 125639. | 3.11-dihydroxyhexadecanoic acid  PubChem CID: 170969. |
| 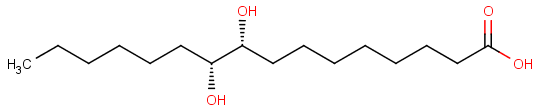 | 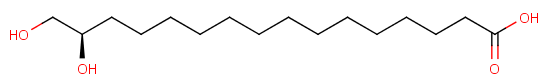 |
| 9.10-dihydroxyhexadecanoic acid  PubChem CID: 193113. | 15.16-dihydroxyhexadecanoic acid  PubChem CID: 5282925. |
