# Supplementary material for: AMPK modulatory activity of olive–tree leaves phenolic compounds: Bioassay-guided isolation on adipocyte model and in silico approach
Source: PLoS One. 2017 Mar 9;12(3):e0173074. doi: 10.1371/journal.pone.0173074 (PMC5344353; doi:10.1371/journal.pone.0173074)

**AMPK modulatory activity of olive**–**tree leaves phenolic compounds: bioassay-guided isolation on adipocyte model and in silico approach**

Cecilia Jiménez–Sánchez^1,2^, Mariló Olivares–Vicente^3^, Celia Rodríguez–Pérez^1,2^, María Herranz–López^3^, Jesús Lozano–Sánchez^1,2^, Antonio Segura–Carretero^1,2^, Alberto Fernández–Gutiérrez^1,2^, José Antonio Encinar^3¶^, Vicente Micol^3,4*¶^

^1^ Department of Analytical Chemistry, University of Granada. Granada, Spain.

^2^ Research and Development of Functional Food Centre (CIDAF), PTS, Granada, Spain.

^3^ Instituto de Biología Molecular y Celular (IBMC), Miguel Hernández University (UMH), Elche, Alicante, Spain

^4^ CIBER: CB12/03/30038, Fisiopatología de la Obesidad y la Nutrición, CIBERobn, Instituto de Salud Carlos III (ISCIII), Palma de Mallorca, Spain.

* Corresponding author

Email: [vmicol@umh.es](mailto:vmicol@umh.es) (VM)

^¶^ These authors share co-senior authorship.

**Supporting information**


S2 Fig. Base peak chromatogram (BPC) made by HPLC-ESI-TOF. This BPC was obtained in negative ion mode of the olive leaf extract, with the optimized semi-preparative conditions, and the collected fractions highlighted in blue.


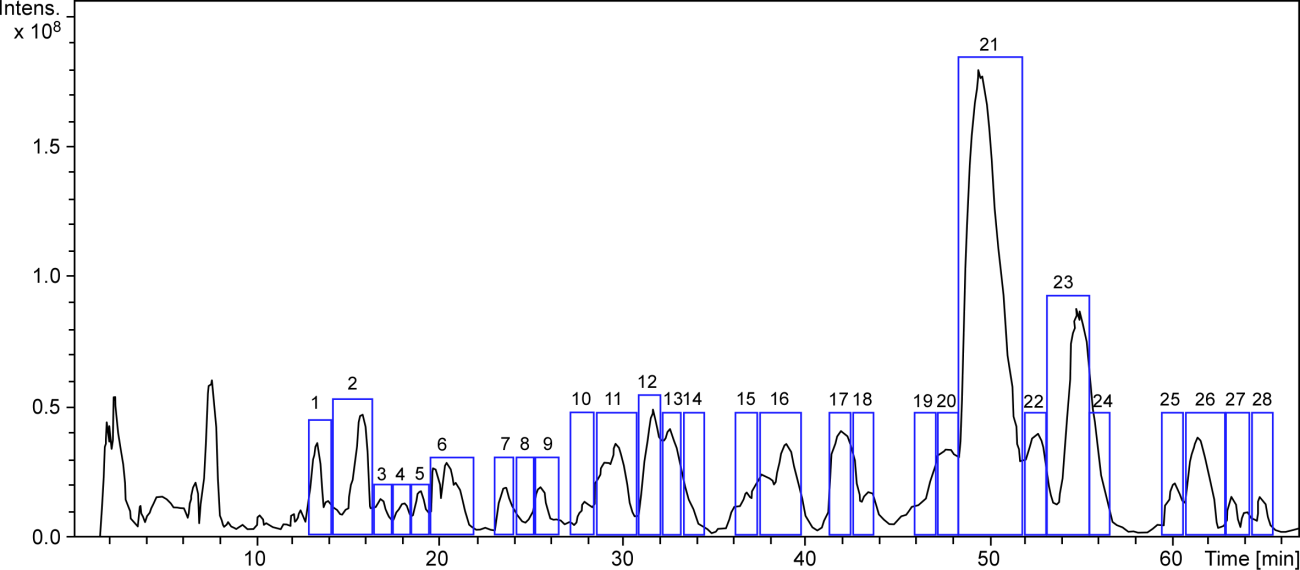

Supplement: S2 Fig — This BPC was obtained in negative ion mode of the olive leaf extract, with the optimized semi-preparative conditions, and the collected fractions highlighted in blue. (DOCX) [file pone.0173074.s002.docx]
